# Consensus guideline for the diagnosis and treatment of tetrahydrobiopterin (BH_4_) deficiencies

**DOI:** 10.1186/s13023-020-01379-8

**Published:** 2020-05-26

**Authors:** Thomas Opladen, Eduardo López-Laso, Elisenda Cortès-Saladelafont, Toni S. Pearson, H. Serap Sivri, Yilmaz Yildiz, Birgit Assmann, Manju A. Kurian, Vincenzo Leuzzi, Simon Heales, Simon Pope, Francesco Porta, Angeles García-Cazorla, Tomáš Honzík, Roser Pons, Luc Regal, Helly Goez, Rafael Artuch, Georg F. Hoffmann, Gabriella Horvath, Beat Thöny, Sabine Scholl-Bürgi, Alberto Burlina, Marcel M. Verbeek, Mario Mastrangelo, Jennifer Friedman, Tessa Wassenberg, Kathrin Jeltsch, Jan Kulhánek, Oya Kuseyri Hübschmann, on behalf of the International Working Group on Neurotransmitter related Disorders (iNTD)

**Affiliations:** 1 Division of Child Neurology and Metabolic Disorders, University Children’s Hospital, Heidelberg, Germany; 2 Pediatric Neurology Unit, Department of Pediatrics, University Hospital Reina Sofía, IMIBIC and CIBERER, Córdoba, Spain; 3 Inborn errors of metabolism Unit, Institut de Recerca Sant Joan de Déu and CIBERER-ISCIII, Barcelona, Spain; 4 Unit of Pediatric Neurology and Metabolic Disorders, Department of Pediatrics, Hospital Germans Trias i Pujol, and Faculty of Medicine, Universitat Autònoma de Barcelona, Badalona, Spain; 5 Department of Neurology, Washington University School of Medicine, St. Louis, USA; 6 Department of Pediatrics, Section of Metabolism, Hacettepe University, Faculty of Medicine, 06100 Ankara, Turkey; 7 Developmental Neurosciences, UCL Great Ormond Street-Institute of Child Health, London, UK; 8 Department of Neurology, Great Ormond Street Hospital, London, UK; 9 Unit of Child Neurology and Psychiatry, Department of Human Neuroscience, Sapienza University of Rome, Rome, Italy; 10 Neurometabolic Unit, National Hospital, Queen Square, London, UK; 11 Department of Pediatrics, AOU Città della Salute e della Scienza, Torino, Italy; 12 Department of Paediatrics and Adolescent Medicine, First Faculty of Medicine, Charles University and General University Hospital in Prague, Prague, Czech Republic; 13 First Department of Pediatrics of the University of Athens, Aghia Sofia Hospital, Athens, Greece; 14 Department of Pediatric, Pediatric Neurology and Metabolism Unit, UZ Brussel, Brussels, Belgium; 15 Department of Pediatrics, University of Alberta Glenrose Rehabilitation Hospital, Edmonton, Canada; 16 Clinical biochemistry department, Institut de Recerca Sant Joan de Déu, CIBERER and MetabERN Hospital Sant Joan de Déu, Barcelona, Spain; 17 Department of Pediatrics, Division of Biochemical Genetics, BC Children’s Hospital, University of British Columbia, Vancouver, BC Canada; 18 Division of Metabolism, University Children’s Hospital Zurich, Zürich, Switzerland; 19 Clinic for Pediatrics I, Medical University of Innsbruck, Anichstr 35, Innsbruck, Austria; 20 U.O.C. Malattie Metaboliche Ereditarie, Dipartimento della Salute della Donna e del Bambino, Azienda Ospedaliera Universitaria di Padova - Campus Biomedico Pietro d’Abano, Padova, Italy; 21 Departments of Neurology and Laboratory Medicine, Alzheimer Centre, Radboud University Medical Center, Donders Institute for Brain, Cognition and Behaviour, Nijmegen, The Netherlands; 22 UCSD Departments of Neuroscience and Pediatrics, Rady Children’s Hospital Division of Neurology; Rady Children’s Institute for Genomic Medicine, San Diego, USA

**Keywords:** Tetrahydrobiopterin deficiency, BH_4_, Neurotransmitter, Guanosine triphosphate cyclohydrolase deficiency, 6-pyruvoyltetrahydropterin synthase deficiency, Sepiapterin reductase deficiency, pterin-4-alpha-carbinolamine dehydratase deficiency, Dihydropteridine reductase deficiency, Hyperphenylalaninemia, iNTD, Consensus guidelines, SIGN

## Abstract

**Background:**

Tetrahydrobiopterin (BH_4_) deficiencies comprise a group of six rare neurometabolic disorders characterized by insufficient synthesis of the monoamine neurotransmitters dopamine and serotonin due to a disturbance of BH_4_ biosynthesis or recycling. Hyperphenylalaninemia (HPA) is the first diagnostic hallmark for most BH_4_ deficiencies, apart from autosomal dominant guanosine triphosphate cyclohydrolase I deficiency and sepiapterin reductase deficiency. Early supplementation of neurotransmitter precursors and where appropriate, treatment of HPA results in significant improvement of motor and cognitive function. Management approaches differ across the world and therefore these guidelines have been developed aiming to harmonize and optimize patient care. Representatives of the International Working Group on Neurotransmitter related Disorders (iNTD) developed the guidelines according to the SIGN (Scottish Intercollegiate Guidelines Network) methodology by evaluating all available evidence for the diagnosis and treatment of BH_4_ deficiencies.

**Conclusion:**

Although the total body of evidence in the literature was mainly rated as low or very low, these consensus guidelines will help to harmonize clinical practice and to standardize and improve care for BH_4_ deficient patients.

## Background

### Introduction

**Fig. 1:**
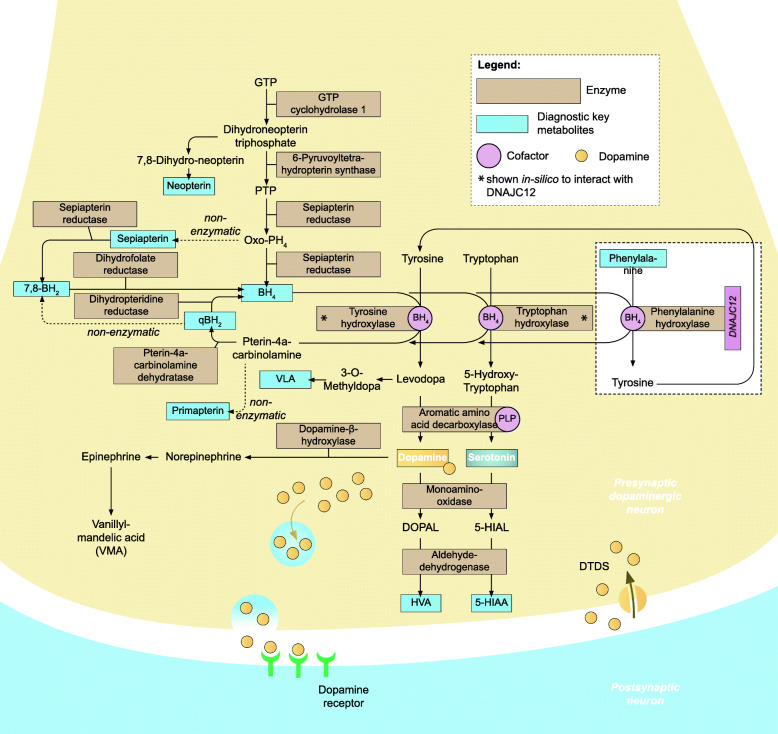
Biosynthesis and regeneration of tetrahydrobiopterin (BH_4_) and its functions as cofactor in the synthesis of serotonin, dopamine, and other catecholamines as well as the catabolism of phenylalanine. Simplified scheme of the biosynthesis and regeneration of tetrahydrobiopterin (BH_4_) in the presynaptic axonal end. BH_4_ serves as essential cofactor of the aromatic amino acid hydroxylases phenylalanine hydroxylase (PAH), tyrosine hydroxylase (TH), and tryptophan hydroxylase (TPH) which catalyse key reactions in the synthesis of the monoamines dopamine, serotonin, norepinephrine, and epinephrine. Note that AGMO and NOSs are not depicted in this overview. 5-HIAA, 5-hydroxyindoleacetic acid; 5-HIAL, 5-hydroxyindoleacetaldehyde; 7,8-BH_2_, 7,8-dihydrobiopterin; BH_4_, tetrahydrobiopterin; DOPAC, 3,4-dihydroxyphenylacetic acid; DOPAL, 3,4-dihydroxyphenylacetaldehyde; DTDS, dopamine transport deficiency syndrome; GTP, guanosine-5′-triphosphate; HVA, homovanillic acid; Oxo-PH41, oxo-2-hydroxy-tetrahydropterin; PLP, pyridoxal 5′-phosphate; PTP, 6-pyruvoyltetrahydropterin; qBH_2_, quinonoid dihydrobiopterin; VLA, vanillyllactic acid; VMA, vanillylmandelic acid; VMAT 2, vesicular monoamine transporter

Tetrahydrobiopterin (BH_4_) deficiencies comprise a group of six rare neurometabolic disorders caused by pathogenic variants in five genes responsible for the biosynthesis and regeneration of BH_4_, which is the essential cofactor of the aromatic amino acid hydroxylases phenylalanine hydroxylase (PAH), tyrosine hydroxylase (TH), two isoforms of tryptophan hydroxylase (TPH 1/2), alkylglycerol mono-oxygenase (AGMO), as well as of three isoforms of nitric oxide synthase (NOS 1–3) (Fig. 1). Since TH and TPH are key enzymes in the synthesis of the monoamines dopamine, serotonin, norepinephrine, and epinephrine, a disturbance of BH_4_ metabolism results in a severe depletion of all monoamine neurotransmitters. In addition, as PAH mediates the conversion of phenylalanine (Phe) to tyrosine (Tyr), hyperphenylalaninemia (HPA) is present in all BH_4_ deficiencies apart from autosomal dominant guanosine triphosphate cyclohydrolase I deficiency (AD-GTPCHD) and sepiapterin reductase deficiency (SRD) [1, 2]. AD-GTPCHD is the most common cause of dopa-responsive dystonia (DRD), a clinical syndrome characterized by dystonia that fluctuates diurnally and responds very well to treatment with levodopa (L-Dopa). AD-GTPCHD is also called autosomal dominant Segawa syndrome (DYT5a), whereas autosomal recessive Segawa syndrome is usually caused by autosomal recessive mutations of the *TH* gene (DYT5b).

BH_4_ synthesis and regeneration is a multistage process involving a series of steps catalysed by five enzymes. Guanosine triphosphate cyclohydrolase I (GTPCH, EC 3.5.4.16), 6-pyruvoyltetrahydropterin synthase (6-PTPS, EC 4.2.3.12), and sepiapterin reductase (SR, EC 4.1.1.17) are the enzymes for BH_4_ biosynthesis. Pterin-4-alpha-carbinolamine dehydratase (PCD, EC 4.2.1.96) and q-dihydropteridine reductase (DHPR, EC 1.5.1.34) ensure BH_4_ regeneration (Fig. 1). All the disorders are inherited in an autosomal recessive (AR) manner, apart from GTPCH deficiency (GTPCHD), which manifests with both autosomal recessive and autosomal dominant (AD) inheritance patterns (Table 1).

**Table 1:** Nomenclature of BH_4_ disorders

Disease name	Alternative disease name	Disease abbreviation	Gene symbol	Mode of inheritance	Affected enzyme	Disease OMIM#
Autosomal dominant GTP cyclohydrolase I deficiency	Segawa disease Dopa-responsive dystonia	AD-GTPCHD, DYT5a	*GCH1*	AD	GTPCH I	128230
Autosomal recessive GTP cyclohydrolase I deficiency	–	AR-GTPCHD, DYT/PARK-*GCH1*	*GCH1*	AR	GTPCH I	233910
6-pyruvoyl-tetrahydropterin synthase deficiency	–	PTPSD, DYT/PARK-*PTS*	*PTS*	AR	PTPS	261640
Sepiapterin reductase deficiency	–	SRD, DYT/PARK-*SPR*	*SPR*	AR	SR	612716
Q-dihydropteridine reductase deficiency	–	DHRPD, DYT/PARK-*QDPR*	*QDPR*	AR	DHPR	261630
Pterin-4-alpha-carbinolamine dehydratase deficiency	Primapterinuria	PCDD	*PCBD1*	AR	PCD	264070

*Abbreviations in the table*: *AR* Autosomal recessive, *AD* Autosomal dominant, *DHPRD* Dihydropteridine reductase deficiency, *GCH1* GTP cyclohydrolase 1, *GTPCHD* Guanosine triphosphate cyclohydrolase I deficiency, *PCBD1* Pterin-4 alpha-carbinolamine dehydratase, *PCDD* Pterin-4-alpha-carbinolamine dehydratase deficiency, *PTPSD* 6-pyruvoyl-tetrahydropterin synthase deficiency, *PTS* 6-Pyruvoyltetrahydropterin synthase, *QDPR* Quinoid dihydropteridine reductase, *SR* Sepiapterin reductase, *SRD* Sepiapterin reductase deficiency

The precise global prevalence of BH_4_ deficiencies remains unknown and great variance can be found among different countries [3, 4]. The mean incidence of all HPAs detected by newborn screening (NBS) programmes in Europe is estimated to be approximately 1:10000 [5], and BH_4_ deficiencies are presumed to constitute around 1–2% of these cases. PTPS deficiency (PTPSD) is the most frequent of all HPA -associated BH_4_ deficiencies (approx. 54%), followed by DHPR deficiency (DHPRD, approx. 33%) [3]. For AD-GTPCHD a prevalence of 2.96 per million was stated [6], however, since many publications do not clearly classify the disease into DRD, AD or AR Segawa syndrome, and do not always mention the underlying gene mutations, a final assessment of the prevalence is not possible [7]. There seems to be a high rate of undiagnosed patients [8-10]. Recently, a novel disorder to be included in the differential diagnosis of HPA has been identified: the disorder is caused by biallelic mutations in the *DNAJC12* gene and is associated with a variable neurological phenotype in association with HPA [11, 12].

Both laboratory and clinical findings in patients with BH_4_ deficiencies are attributable to two main pathophysiologic mechanisms: HPA, and depletion of the monoamine neurotransmitters in the central nervous system (CNS).

Cerebral HPA toxicity is multifactorial. The most prominent hypotheses discussed include: 1) competitive inhibition of a blood-brain-barrier (BBB) transporter of large neutral amino acids (LNAA) including tyrosine and tryptophan with decreased protein and neurotransmitter synthesis; 2) decreased cholesterol synthesis and myelin production, as well as direct myelin toxicity; 3) tyrosine and tryptophan hydroxylase inhibition; 4) oxidative stress; 5) complex reduction of glutamatergic synaptic transmission; 6) pyruvate kinase inhibition; 7) calcium homeostasis dysregulation [5, 13].

The second, and clinically dominant, pathophysiological mechanism of neurological dysfunction in the BH_4_ deficiencies is shortage of the brain neurotransmitters dopamine, serotonin, and norepinephrine. Dopamine is most commonly associated with the control of voluntary movement and reward-based learning and behaviour [14]. Norepinephrine is the modulator of arousal [15] and serotonin affects predominantly higher cognitive functions and behaviour. However, deeper insight into the complexity of CNS neurotransmission reveals that monoaminergic neurons and their neurotransmitters share many common properties, greatly overlap in numerous functions, and are highly orchestrated to jointly modulate numerous brain processes. As a result, dopamine, serotonin, and norepinephrine are all implicated in the modulation of higher cognitive and executive function, behaviour, mood, attention, pain perception, motor control, and many other brain processes [16]. Attribution of any given clinical symptom to the deficiency of a single neurotransmitter is therefore likely to be an over-simplification. However, the general signs of dopamine deficiency include predominantly parkinsonism, and dystonic movements, in young infants also tremorous or choreatiform and various other involuntary movements, while serotoninergic deficiency is thought to manifest as sleep pattern disturbance, mood dysregulation and temperature instability [17].

Following the two main underlying pathophysiologic pathways, treatment strategies primarily aim at the correction of peripheral HPA and brain neurotransmitter deficiencies. We developed this first consensus guideline for the diagnosis and management of BH_4_ deficiencies in the context of the International Working Group on Neurotransmitter Related Disorders (iNTD, www.intd-online.org) and by using the Scottish Intercollegiate Guideline Network (SIGN) methodology. The recommendations are based on a systematic review of the available literature and on consensus meetings of the iNTD guideline working group. The guideline is intended for metabolic specialists, child and adult neurologists, paediatricians, intensive care specialists, nurses, and paramedical specialists involved in the care of patients with BH_4_ deficiencies.

## Methods

### Composition of the guideline working group and timeline

An executive committee (TO (chairman), OKH (secretary, subgroup coordinator), ELL, ECS, JK (subgroup coordinators) and KJ (project coordination)) was appointed to oversee the guideline development process. Four different subgroups were generated, headed by one of the subgroup coordinators (AR/AD GTPCHD (ELL), PTPSD (JK), DHPRD (ECS) and PCDD/SRD (OKH)). The guideline working group consisted of 24 child neurologists and/or metabolic specialists (TO, ELL, ECS, TP, SSB, BA, MK, VL, WL, FP, AGC, TH, RP, LR, HG, GFH, GH, SBB, AB, MM, JF, TW, JK, OKH), 5 biochemists (RA, BT, SH, SP, MV), and 1 research project manager (KJ) from several European countries, the USA and Canada. All group members are part of the iNTD network and experienced in the diagnosis and treatment of BH_4_ deficiencies. The project was supported by patient organizations (see below, section on “Patient advocacy groups”).

The start-up meeting took place in Barcelona, Spain in February 2017, followed by one meeting of the subgroup coordinators in Heidelberg, Germany in November 2017 and a face-to-face meeting for the whole guideline group in Athens, Greece in September 2018 (at the meeting of the Society for the Study of Inborn Errors of Metabolism (SSIEM)).

In adherence to Scottish Intercollegiate Guidelines Network (SIGN) methodology, two external academic reviewers with expertise in neurometabolic and movement disorders (Nicola Longo, Salt Lake City, USA and Keith Hyland, Atlanta, USA) and additional lay reviewers (Pauline Schleicher, Melanie Kahlo and Ivana Badnjarevic) were asked to comment on the guideline draft before submission.

### Developing topics and key questions

During the start-up meeting, the list of key questions was discussed and refined. Key questions comprised the following topics: Clinical Presentation, Diagnosis (laboratory tests, imaging, electrophysiological investigations, etc.), Treatment, Management of Complications and Long-Term Follow-Up, Social Issues, and Transition (Additional file 1: Table S1). All topics were considered by each of the 4 different BH_4_ disorder subgroups.

### Systematic literature search

A systematic literature review on BH_4_ and related names was performed in spring 2017 on Pubmed, Cochrane database, and the Cinahl database, using the following search terms: “Tetrahydrobiopterin deficiency”, “BH_4_ deficiency”, “atypical PKU”, “atypical phenylketonuria”, “PTPS deficiency”, “(6-)pyruvoyl-tetrahydropterin synthase deficiency”, “SR deficiency”, “sepiapterin reductase deficiency”, “segawa(s) disease”, “GTPCH (I) deficiency”, “GTP cyclohydrolase (I) deficiency”,“Guanosine (-5-) triphosphate cyclohydrolase (I) deficiency”,” DHPR deficiency”, “dihydropteridine reductase deficiency”, “pterin (-4a-) carbinolamine dehydratase deficiency”, “PCD deficiency“. No language or data filters were used. Single newly published manuscripts with clear clinical relevance for the guideline development were included in the literature database until the end of guideline development process. Reference lists from review articles and key case series were screened for additional hits and members of the guideline group were asked to suggest relevant book chapters. The flow chart in the supplementary material illustrates the literature search (Additional file 2: Figure S2).

### Grading of evidence and recommendations

The guideline was developed according to methodology of the SIGN [18]. For rating the quality of evidence and defining the strength of recommendations, SIGN is committed to using the Grading of Recommendations, Assessment, Development and Evaluation (GRADE) methodology. The level of evidence of individual studies was rated from 4 (lowest) to 1++ (highest). Specific outcomes (e.g. effect of a specific drug on hypotonia) were described in relation to the quality of evidence (very low, low, moderate or high) for the total body of evidence. Recommendations were rated as strong (for or against), conditional (for or against), or classified with a recommendation for further research (Table 2).

**Table 2:** Forms of recommendations

Judgment	Recommendation
Undesirable consequences clearly outweigh desirable consequences	Strong recommendation against
Undesirable consequences probably outweigh desirable consequences	Conditional recommendation against
Balance between desirable and undesirable consequences is closely balanced or uncertain	Recommendation for research and possibly conditional recommendation for use restricted to trials
Desirable consequences probably outweigh undesirable consequences	Conditional recommendation for
Desirable consequences clearly outweigh undesirable consequences	Strong recommendation for

Furthermore, Good Practice Points (GPP) were formulated based on the clinical experience of the guideline development group. Relevant papers were evaluated by at least two guideline working group members. Before and during meetings, the guideline group members were trained in standardized literature evaluation using SIGN/GRADE methodologies. All recommendations were discussed for consensus during guideline group meetings.

### Disclaimer

The purpose of this guideline is to improve care for patients with BH_4_ deficiencies. It is not intended to replace sensible, well-informed clinical care. Although the guideline is based on the best available evidence, the body of evidence for these disorders is comprised mainly of non-analytical studies and case reports. In addition, some recommendations reflect expert, often consensus opinion. Nevertheless, we believe that this guideline, which is meant to provide a solid foundation to caregivers of BH_4_ deficient patients, will improve the care for these patients around the world.

## Clinical presentation

The data on the clinical phenotype of BH_4_ deficiencies (BH_4_Ds) was collected from a retrospective analysis of published case reports. The level of precision and proficiency regarding the recognition and the use of medical terminology to describe clinical symptoms varied substantially among individual publications, resulting in a certain level of imprecision. After the establishment of the first registry on BH_4_Ds with HPA which gathered clinical, biochemical, and treatment data (Database of Patients and Genotypes Causing HPA/Phenylketonuria (PKU) incl. BH_4_-Responsive Phenotype, BIODEF; http://www.biopku.org/home/biodef.asp), more precise information on case series could be obtained for AR-GTPCHD, PTPSD, DHPRD and PCDD [3, 19]. Since 2015, the International Working Group on Neurotransmitter related Disorders (iNTD; http://www.iNTD-online.org) provides the first patient registry for all neurotransmitter - related disorders with a standardized longitudinal assessment of complex patient data [20].

### General clinical pattern of BH_4_Ds and differential diagnosis

The cardinal symptoms of BH_4_Ds reflect dopamine deficiency as well as the imbalance of other neurotransmitters including serotonin, norepinephrine or epinephrine in the CNS (Table 3). While the overall clinical phenotype of BH_4_Ds may overlap with numerous other disorders, e.g. cerebral palsy [21], certain clinical features may raise the clinical suspicion for a disorder of impaired neurotransmission (e.g. early onset parkinsonism, oculogyric crises, diurnal fluctuation of symptoms, or an unexplained cerebral palsy-like picture). It is important to note that patients may show a wide spectrum of clinical severity, ranging from asymptomatic individuals requiring no treatment to very severe disease courses.

**Table 3:** Symptoms and signs described in the different BH_4_ deficiencies

	PTPSD	DHPRD	AR-GTPCHD	SRD	PCDD	AD-GTPCHD
Number of reported cases	125	77	55	53	19	570
**Nervous system**						
Developmental delay	+++	+++	+++	+++	+	(+)
(Axial) Hypotonia	+++	++	+++	+++	+	(+)
Poor head control	+	+	+	+		(+)
Hypertonia	++	++	++	++	(+)	++
Epilepsy	++	+++	+	+		
Cognitive impairment	++	+	(+)	++		(+)
Impaired speech development	+	(+)	(+)	+++		(+)
Dysarthria	(+)	(+)		+++		(+)
**Movement disorders**						
Diurnal fluctuation of symptoms			+	+++		+++
Dystonia	+	+	++	+++		+++
Oculogyric crises	(+)	+	(+)	+++		(+)
Gait difficulties						+++
Dyskinesia/other involuntary movements	+	+	(+)	+/++		
Parkinsonism/hypokinesia	+	(+)/+	+	+++		+
Tremor	(+)	(+)	(+)		(+)	+
Ataxia	(+)	(+)		+		(+)
**Other**						
Hyperreflexia	(+)/+	+				++
Irritability	(+)/+	+				
Microcephaly		++	(+)	(+)		
**Autonomous nervous system**						
Temperature instability	(+)	(+)	++	++		
**Gastrointestinal system**						
Hypersalivation	(+)	+	++	+/++		
Feeding/swallowing difficulties	+	+	(+)	++		(+)
**Psychological problems**						
Behavioural problems	(+)	(+)		(+)		
Psychiatric problems	(+)	(+)		++		+
**Sleep problems**	(+)	(+)		++		(+)
**Endocrine disturbances**						
Growth-hormone deficiency	(+)					
Low birth weight	++					
Central hypothyroidism				(+)		
MODY3-like diabetes					+	
**Other**						
Microcephaly		++	(+)	(+)		
Fatigability	(+)	+		(+)		(+)
Recurrent chest infections	+					
Prematurity	+	+				
Hypomagnesemia					+	

Symptoms and signs reported in 570 Patients with AD-GTPCHD, 55 patients with AR-GTPCHD, 125 patients with PTPSD, 77 patients with DHPRD, 53 patients with SRD and 19 patients with PCDD

Very frequently +++ (≥50%), frequently ++ (≥25- < 50%), infrequently + (≥10- < 25%), occasionally (+) (< 10%)

### Clinical patterns specific of PTPSD, DHPRD, AR-GTPCHD, and SRD

The most common symptoms prior to treatment initiation were assessed in 125 PTPSD, 77 DHPRD, 55 AR-GTPCHD and 53 SRD patients. Hypotonia, impaired motor development and cognitive development, movement disorders (mainly dystonia), and parkinsonism/hypokinetic rigid syndrome (consisting of bradykinesia, extrapyramidal rigidity (“cogwheel rigidity”), rest tremor and/or postural instability) are the hallmarks of BH_4_Ds and should prompt clinicians to include BH_4_Ds in the differential diagnosis.

Table 3 summarizes frequencies of the individual symptoms: Approximately 50–75% of patients with any of these disorders have hypotonia, often combined with poor head control and peripheral hypertonia, mainly of the extremities. Developmental delay of variable severity is another common symptom, described in > 50% of patients. Specific impairment of cognitive and speech development was reported in around 50% of SRD and PTPSD patients and in substantially lower proportions (5–15%) in the remaining BH_4_Ds. However, it should be noted that in most studies, standardized neuropsychological assessment is lacking, so that there is a risk of over- or underestimating cognitive function.

Movement disorders, mainly dystonia, have been reported in almost 60% of patients with SRD, but less frequently in other BH_4_Ds (10–35%). Oculogyric crises were reported in 60% of patients with SRD and only in 5–15% of patients with DHPRD, AR-GTPCHD and PTPSD. It is important to note that the SRD cohort is comprised of patients whose clinical features have been very precisely characterized. Therefore, it should be kept in mind that these symptoms, as well as those described below, may be just as common or even more common in the BH_4_Ds other than SRD, but underestimated in published reports where patients’ clinical phenotypes were less precisely characterized.

Dyskinesia or other types of involuntary movements such as rest/postural tremor were not frequent and occurred predominantly in SRD patients. Ataxia is not often reported in any of the BH_4_D patient groups.

Early-onset parkinsonism or hypokinetic rigid syndrome is associated with only a limited differential diagnosis in infancy and childhood, and should prompt clinicians to include investigations for BH_4_Ds in the diagnostic approach. Parkinsonism or hypokinetic rigid syndrome was identified in around 60% of SRD, 25% of PTPSD, and 10% of DHPRD and AR-GTPCHD patients. Diurnal fluctuation of movement symptoms (worsening over the course of the day; subsequent improvement after rest) is generally regarded as characteristic of neurotransmitter disorders. It was, however, reported in 68% of patients with SRD and only around 10% of patients with AR-GTPCHD.

Patients with DHPRD in particular may develop epileptic seizures, while seizures are less common in patients with PTPSD, and occur rarely in SRD and AR-GTPCHD. No specific type of epilepsy was identified. Interestingly, roughly 10% of patients with PTPSD and DHPRD were reported to have irritability and hyperreflexia, while these symptoms were reported in neither SRD nor AR-GTPCHD.

Autonomic dysregulation, reflecting the disruption of neurotransmitter homeostasis and most frequently manifesting as temperature instability, was documented primarily in SRD and AR-GTPCHD (almost 35%). Excessive salivation was regularly reported in all BH_4_D, again most commonly in AR-GTPCHD and SRD (25–40%), and less frequently in DHPRD and PTPSD (5–15%). Swallowing/feeding difficulties, presumably due to overall motor impairment and/or oropharyngeal dystonia, were seen in 20 to 30% of patients with SRD, PTPS and DHPRD.

Single case reports add endocrine dysfunction like growth-hormone deficiency (PTPSD) or central hypothyroidism (SRD) to the clinical phenotype [22, 23]. An increased frequency of prematurity has been reported in PTPSD and DHPRD. In PTPSD, a tendency towards low birth weight has been observed [3]. Up to 25% of patients with DHPRD were reported to be microcephalic, as opposed to the other disorders, in which microcephaly was reported in only 1% of patients.

Various psychiatric and behavioural problems (including depression, anxiety, psychosis, obsessive compulsive features, impulsivity, and attention deficit disorder) as well as sleep disturbance are reported infrequently in all of the BH_4_Ds. However, psychiatric and behavioural problems are likely underdiagnosed, except in SRD, where they were documented in around 45% of patients [21, 24–28].

**R#1: (strong):** In patients with unexplained alterations in muscle tone (hypotonia/hypertonia), movement disorders (dystonia, oculogyric crises), parkinsonism or hypokinetic rigid syndrome, autonomic dysfunction or diurnal fluctuation of symptoms, BH_4_Ds should be considered.

**R#2: (strong):** Clinical follow-up should include the assessment of psychiatric or behavioural problems and sleep disorders.

### Clinical patterns specific to PCDD

PCDD represents the rarest BH_4_D. A clear clinical description has been documented in the literature in only 19 patients (Table 3). In the BIODEF database most patients were reported to be asymptomatic (as of 01.05.2019), although a few patients exhibited transient alterations in muscle tone, slight tremor or transient and very mild delay in motor development [29]. However, mutations in *PCBD1* are associated with both hypomagnesaemia and risk for HNF1A-like Maturity Onset Diabetes of the Young (MODY3) diabetes in puberty [30], so patients with *PCBD1* mutations should be screened for these disorders.

**R#3: (strong)**: Patients with PCDD should be screened for hypomagnesaemia and the development of HNF1A-like MODY3 diabetes during puberty.

### Specific clinical pattern of AD-GTPCHD

Data regarding the clinical spectrum of AD-GTPCHD prior to treatment initiation was reviewed in 570 patients (Table 3). Clinical symptoms in AD-GTPCHD differ substantially in many aspects from the remaining BH_4_Ds. The phenotype is milder and in more than 50% of cases dominated by postural or action-induced dystonia of one or both lower limbs manifesting as gait difficulties. Diurnal fluctuation of motor symptoms, with worsening later in the day, is a very characteristic finding in AD-GTPCHD, especially during the first 3 decades [9]. Later, fluctuations become less prominent. If not treated, focal or segmental dystonia typically progresses to multifocal or even generalized dystonia (observed in 15%), together with the development of parkinsonian signs in some cases (reported in 13%). The clinical presentation in the second decade of life is characterized by action dystonia of the upper limbs, sometimes associated with cervical impairment, asymmetric tremor and parkinsonism [8]. After the age of 20 years the predominant (> 80%) presentation is parkinsonism (isolated or combined with dystonia). The progression of the dystonia (in both symptom severity and spread of symptoms to previously unaffected body parts) and the diurnal fluctuation of symptoms subside with age and the disease becomes almost stable in the fourth decade. Increased risk of typical degenerative parkinsonism has been reported in adulthood with rare *GCH1* variants [31]. Psychiatric disorders have been reported in 10% of patients. Other symptoms observed in the recessive forms of BH_4_D, such as hypotonia, developmental delay, cognitive impairment, oculogyric crises or epilepsy, occur extremely rarely in patients with AD-GTPCHD.

**R#4: (strong):** In patients with dystonia, especially of the lower limbs, with onset in the first or second decade of life associated with diurnal fluctuation of symptoms and normal development, possibly accompanied by parkinsonism, AD-GTPCHD should be considered.

### Age of onset and age of diagnosis

Precise data on the age of disease onset and the age of diagnosis could not be reliably gathered from a retrospective analysis of published case reports. However, up to 40% of patients with BH_4_Ds can be asymptomatic during the neonatal period. With increasing age, the percentage of asymptomatic patients decreases significantly (except PCDD) [3].

BH_4_Ds presenting with HPA can be detected by NBS and are therefore diagnosed early (between 2 and 14 days of life) [3]. The absence of HPA in SRD markedly delays the diagnosis (mean age at diagnosis 8.9 years), although the first symptoms may be apparent as early as within the first 18 months of life [3, 21, 27, 32]. In AD-GTPCHD patients, disease onset is typically during the first decade of life (mainly between 3 to 9 years of age), although very rarely patients may present with dystonia and/or developmental delay in the first 12–18 months [33, 34]. Disease onset in the second decade is also common. The average delay in diagnosis (in the time before easily accessed diagnostic whole exome sequencing) has been stated to be around 10 years [10].

### Phenotype correlations with genotype or biochemical phenotype

There are many different variants described in all BH_4_D genes (Table 1). The detailed list is constantly updated (see https://www.ncbi.nlm.nih.gov/clinvar/, http://www.hgmd.cf.ac.uk or https://omim.org, search term “tetrahydrobiopterin” assessed December, 2019) and is beyond the scope of this guideline project.

For PCDD, AR-GTPCHD, DHPRD, and PTPSD, there are no consistent reports on genotype–phenotype correlation. For AD-GTPCHD, there is certain heterogeneity: Some publications discuss and exclude genotype-phenotype correlation [35, 36]. Others describe different large heterozygous *GCH1* deletions with high penetrance and association with multifocal dystonia and adult onset in a Taiwanese DRD population [37]. In 43 patients with SRD with 16 different *SPR* mutations, no clear genotype–phenotype correlation was documented [21].

## Diagnosis: laboratory tests

### Key diagnostic test: newborn screening

The pattern of increased Phe concentrations with reduced Tyr concentrations resulting in elevated phenylalanine/tyrosine ratio detects all forms of HPA in national newborn screening (NBS) programs [38]. Among the BH_4_ disorders, AR-GTPCHD, PTPSD, DHPRD and PCDD classically present with HPA. Detailed results of NBS are available from 15 AR-GTPCHD, 305 PTPSD, 46 DHPRD, and 18 PCDD cases. Patients with AR-GTPCHD can be missed on NBS due to missing HPA [19, 39, 40], while those published cases where HPA on NBS in DHPRD was not detected, are more likely due to unreliable (historical) methods of Phe detection (e.g. Phenistix [41, 42];). In comparison, NBS was negative in two cases of PTPSD. However, retrospective re-evaluation revealed that the analysis was most likely done by the semiquantitative bacterial inhibition assay (Guthrie method), known to cause false-negative results [43, 44]. In PCDD, all reported cases presented with HPA. The level of HPA in NBS can vary widely and is not indicative of a specific BH_4_D. PCDD tends to be associated with lower Phe levels. No correlation has been observed between the NBS Phe level and the subsequent disease course.

Today, levels of Phe and Tyr are measured by tandem mass-spectrometry (MS). The measurement of Phe in dried blood spot (DBS) is a temperature and light stable method, and available in many countries worldwide.

**R#5 (strong):** Newborn screening for PKU should be performed in all countries following standardized protocols and using modern laboratory techniques to identify elevated levels of Phe. Detection of HPA may be the first clue for underlying AR-GTPCHD, PTPSD, DHPRD or PCDD.

**R#6 (strong):** NBS is not a suitable diagnostic tool for AD-GTPCH and SRD.

**R#7 (GPP):** Patients diagnosed with HPA on NBS should be referred to a specialized metabolic centre for further diagnostic evaluation and prompt initiation of treatment.

### Key diagnostic test: blood phenylalanine (plasma/serum)

As in DBS, increased Phe concentrations in plasma point to all forms of HPA [38]. Although the measurement of Phe concentrations in DBS with MS has several advantages over plasma analysis (easier to obtain and transport, minimal sample preparation, stable metabolites in DBS), there is evidence that Phe quantification in plasma is more precise [45]. Comparison studies of simultaneous measurement of Phe concentration using a MS or an ion-exchange chromatography protocol in either DBS or plasma samples indicated that Phe concentrations were up to 26% lower if measured in DBS [46].

**R#8 (conditional):** Any newborn screening result of HPA should be confirmed by quantification of the Phe level in plasma before treatment is started.

### Key diagnostic test: Pterins in DBS and urine

**Fig. 2:**
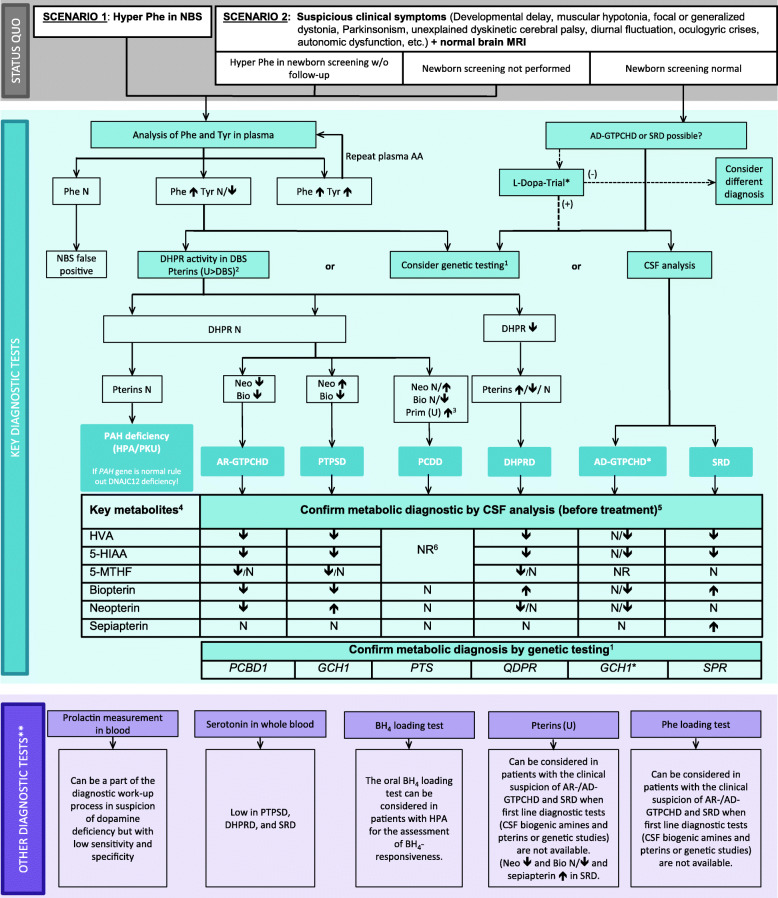
Diagnostic flowchart for differential diagnosis of BH_4_Ds with and without HPA. ^1^Consider genetic HPA workup depending on availability and financial resources. The gene panel should include the *QDPR, GCH1, PTS PCBD1, SPR* genes as well as *DNAJC12.* For GCH1, consider MLPA if Sanger sequencing is negative. ^2^The analysis in urine is more sensitive than in DBS and pathological patterns suggestive for PCDD and SRD can only be detected in urine but not in DBS. ^3^Primapterin measurement in urine is only elevated in PCDD. ^4^Aminoacids in CSF are not required for diagnosis of BH_4_Ds. ^5^CSF analysis should always include standard measurements (cell count, proteins, glucose and lactate). ^6^Recommendation against measurements of HVA, 5-HIAA, 5-MTHF, and pterins in CSF in the case of PCDD. (*) A diagnostic L-Dopa trial should be limited to children with symptoms suggestive of dopa-responsive dystonia or to situations where biochemical and genetic diagnostic tools are not available. If the diagnostic L-Dopa trial is positive but the results of CSF biochemical and/or molecular genetic testing are not compatible with AD-GTPCHD or SRD, further aetiologies for dopa responsive dystonia should be considered (e.g. juvenile parkinsonism (PARK2gene)). (**) Can be considered if available. See text for more detailed information. Abbreviations: 5-HIAA, 5-hydroxyindoleacetic acid; 5-MTHF, 5-methyltetrahydrofolate; AA: amino acids; AD−/AR- GTPCHD: guanosine triphosphate cyclohydrolase I deficiency; BH_4_, tetrahydrobiopterin; Bio: biopterin; CSF: cerebrospinal fluid; DBS: dry blood spot; DHPR: q-dihydropteridine reductase; DHPRD, dihydropteridine reductase deficiency; HVA, homovanillic acid; MRI, magnetic resonance imaging; N: normal; NBS: newborn screening; Neo: neopterin; NR: not reported; PAH: phenylalanine hydroxylase; Phe: phenylalanine; PKU: phenylketonuria; Prim: primapterin; PTPSD, 6-pyruvoyltetrahydropterin synthase deficiency; SRD: sepiapterin reductase deficiency; Tyr: tyrosine; u: urine; (+) = positive effect; (−) = no or no clear effect

Apart from BH_4_Ds, the differential diagnosis of HPA includes phenylalanine hydroxylase (PAH) deficiency, DNAJC12 deficiency, high natural protein intake, prematurity, and liver disease. One option to further investigate the underlying cause of HPA is the analysis of pterins in DBS or urine. With the exception of AD-GTPCHD and DHPRD, each BH_4_D presents with a specific pterin pattern [47] (Fig. 2). AR-GTPCHD reveals low biopterin and neopterin (in DBS and urine). In PTPSD, neopterin is highly elevated along with low biopterin (DBS and urine). In PCDD, primapterin is high in urine, while biopterin has been reported to be low to normal, and neopterin normal to high. Primapterin is not elevated in any other BH_4_D and cannot be reliably detected in DBS. In DHPRD, no consistent pattern of biopterin and/or neopterin levels DBS or urine has been documented: Most patients showed normal neopterin with low to normal biopterin, although a few had normal to elevated neopterin with high biopterin. In some patients both elevated biopterin and neopterin were observed.

In AD-GTPCHD, low to normal values of biopterin and neopterin have been reported in urine [48]. There is no data on DBS. Sepiapterin is typically elevated in SRD, but can be detected in urine only via an additional assay [49]. Biopterin and neopterin are typically normal in DBS and urine in this disorder.

In comparison to the analysis of DBS, the measurement of pterins in urine is more sensitive due to their higher concentrations in urine. On the other hand, the DBS method provides easy sample handling and low transport costs [50]. It should be noted that pterins in urine are more susceptible to degradation by light and high temperature than in DBS. Collection and handling of both urine and DBS should be performed by strictly following standardized procedures to ensure the accuracy of results. Both analyses are available in specialized laboratories, mainly in first world countries.

**R#9 (strong):** Strong recommendation for the analysis of pterins in urine or DBS in patients with HPA on NBS. Pterin analysis cannot rule out DHPRD (see below for enzyme activity measurements).

Be aware that analysis in urine is more sensitive than in DBS, and that pathological patterns suggestive for PCDD and SRD can only be detected in urine. In the case of clinical suspicion, sepiapterin in urine must be requested separately.

Note: Depending on availability and financial resources, a primary genetic HPA workup can be considered (see below).

**R#10 (conditional/research):** Analysis of pterins in urine can be considered in patients with clinical suspicion of AD-GTPCH, where cerebrospinal fluid (CSF) studies or molecular genetic testing are not available.

### Key diagnostic test: DHPR enzyme activity

DHPRD can be reliably detected only by the determination of DHPR activity in DBS [51]. In the literature, DHPR enzyme activity results in DBS were reported in 31 cases with AR-GTPCHD, in 1 case with AD-GTPCHD, 176 cases with PTPSD, 151 cases with DHPRD, and 6 cases with PCDD. The analysis resulted in a reduced DHPR activity only in DHPRD while being normal in the other BH_4_Ds.

Although conclusions drawn from case reports and small case series per se have low levels of evidence, the findings were highly consistent in DHPRD. Of 151 reported patients, 150 had a reduced or even absent DHPR activity in DBS in every laboratory. In one patient, normal DHPR activity was documented. However, the authors in that case consider technical problems to have been highly likely, and reported that the parents did not consent to a repetition of the analysis [52].

Measurement of DHPR activity in DBS is a light and temperature sensitive method. It is available in specialized laboratories, mainly in first world countries.

**R#11 (strong):** Strong recommendation for the analysis of DHPR enzyme activity in DBS in patients with HPA in NBS and/or in case of clinical suspicion of disorders of BH_4_ deficiency.

### Key diagnostic test: lumbar puncture (HVA, 5-HIAA, neopterin, biopterin and sepiapterin and 5-MTHF in CSF)

Secondary to the shortage of the essential cofactor BH_4_ and the consecutively impaired function of the aromatic amino acid hydroxylases, levels of 5-hydroxyindoleacetic acid (5-HIAA) and homovanillic acid (HVA) in CSF are typically significantly low in the BH_4_Ds, apart from PCDD [17]. Notably, normal levels of HVA and 5-HIAA have been documented in published AD-GTPCHD cases (normal HVA in 27% (5 of 18 patients) and normal 5-HIAA in 45% (9 of 20 patients) and in approx. 37–41% of PTPSD cases [25, 53–55], probably representing a milder phenotype. In DHPRD, normal levels of HVA were reported in 9/130 patients while normal 5-HIAA levels were found in 2 patients [24, 56–58]. All patients with SRD had low levels of HVA and 5-HIAA. However, some patients were reported to have initially normal CSF HVA and 5-HIAA levels associated with a mild phenotype, before evolving to a more severe phenotype associated with low CSF HVA and 5-HIAA levels.

Additional evaluation of CSF neopterin, total biopterin or BH_4_, and dihydrobiopterin (BH_2_) makes it possible to biochemically differentiate between the different BH_4_Ds by determining the relevant level of the metabolic block in BH_4_ biosynthesis or regeneration. Both neopterin and biopterin are low in AR-GTPCHD and in most cases of AD-GTPCHD, but in the latter an isolated neopterin decrease seems to be more frequent [59, 60]. High neopterin with low biopterin points to PTPSD. Depending on the analytical method, elevated total biopterin or elevated BH_2_ points to DHPRD or SRD. Sepiapterin is highly elevated in SRD and normal in all other BH_4_Ds. Pterins in CSF are normal in PCDD.

5-methyltetrahydrofolate (5-MTHF) is one of the naturally occurring folates containing a methyl carbon unit attached to the N5 nitrogen atom [61]. The methyl unit in 5-MTHF is essential for various processes in CNS including the methylation of homocysteine to methionine, and the formation of S-adenosyl-methionine (SAM). The latter is required for more than 100 methylation reactions in cells, including methylation of DNA, RNA, neurotransmitters, lipids, hormones, and drug metabolites [61]. Due to the close interplay between pterin and folate metabolism, depletion of 5-MTHF in the CSF can occur in BH_4_Ds. Hereby specifically the DHPR enzyme supports dihydrofolate reductase (DHFR) to maintain folate in its active “tetrahydro” form, in which it is capable of serving as a precursor for the universal methyl donor substance SAM [62].

5-MTHF measurements in CSF were reported in 15 cases with AR-GTPCH, 83 cases with PTPSD, 63 cases with DHPRD and 3 cases with SRD. No reports were available for AD-GTPCH and PCDD. In DHPRD, low levels of 5-MTHF were reported [63] while patients with AR-GTPCH and PTPSD have normal to low levels. 5-MTHF in SRD was normal. In addition, high-dose L-Dopa/carbidopa supplementation can reduce CSF 5-MTHF levels [64].

Analysis of dopamine and serotonin metabolites, pterins, and 5-MTHF is performed in a limited number of specialized laboratories. An online list of iNTD affiliated laboratories is available at the website www.intd-online.org. Collection and analysis of CSF metabolites requires the use of strict protocols and timing to avoid analytical pitfalls [65]. Since normal results of single parameters may be found, CSF analyses should always consist of a combination of monoamines (ideally including 3-*O*-methyldopa (3-OMD), l-3,4-dihydroxyphenylalanine (L-Dopa), 5-hydroxytryptophan (5-HTP)), pterins, and 5-MTHF to ensure the correct interpretation of results by pattern recognition. Patients’ medication at the time of CSF sampling should be documented.

**R#12 (strong):** CSF analysis of HVA, 5-HIAA, pterins and 5-MTHF is a reliable diagnostic method to differentiate between the BH_4_Ds. Specific measurements in CSF should include the core metabolites HVA, 5-HIAA, pterins and 5-MTHF. Pterins can be used to differentiate between different BH_4_Ds.

**R#13 (strong):** Recommendation against measurement of HVA, 5-HIAA, 5-MTHF, and pterins in CSF for PCDD.

**R#14 (GPP):** CSF measurements should always include standard measurements (cell count, protein, glucose, lactate) considering possible differential diagnosis e.g. infection or inflammation of different origin [65]. Collection and handling of CSF should be performed by strictly following standardized procedures to ensure correct interpretation of results.

### Key diagnostic test: genetic testing

For all enzymes involved in the biosynthesis or regeneration of BH_4_, gene variants have been reported in many patients (see https://www.ncbi.nlm.nih.gov/clinvar/). Therefore, mutation detection is the preferred method for diagnosis confirmation or in case of non-conclusive metabolite profiles. All BH_4_Ds are autosomal recessive disorders apart from AD-GTPCHD where heterozygous mutations in the *GCH1* gene cause childhood-onset dopa-responsive dystonia with diurnal fluctuation [66]. In *GCH1,* sequence alterations have been found by Sanger sequencing in only 50 to 60% of clinically typical AD-GTPCHD cases [67]. Since deletions can occur in *GCH1*, the detection requires special methods such as quantitative real-time polymerase chain reaction (qPCR) or multiple ligation-dependent probe amplification (MLPA) [35].

Recently, biallelic mutations in the *DNAJC12* gene, coding for a heat shock co-chaperone of the HSP40 family, have been identified in individuals with mild HPA and a broad spectrum of clinical symptoms including dystonia, speech delay, axial and limb hypertonia, parkinsonism and psychiatric features [12]. Treatment with sapropterin dihydrochloride and/or neurotransmitter precursors L-Dopa/decarboxylase (DC) inhibitor and 5-HTP had beneficial effects and resulted in the prevention of neurodevelopmental delay in individuals treated before the onset of symptoms [11, 12]. Exclusion of *DNAJC12* gene variants has therefore been suggested to be mandatory in all patients with HPA where pterins, DHPR activity and *PAH* gene analysis are normal [11].

DNA (from peripheral blood cells, tissues, cultured cells or dried blood spots) is the preferred sample.

The increasingly broad availability of multi-gene panel testing or next-generation sequencing (NGS) provides a time- and cost-effective approach that will assist clinicians to identify the correct diagnosis in patients with absent biomarkers or atypical clinical features. The identification of disease-causing mutations facilitates accurate prenatal diagnosis, determination of the carrier status of family members, and genetic counselling [68, 69].

**R#15 (strong):** Biochemical diagnosis of BH_4_Ds should be confirmed by molecular genetic analysis.

**R#16 (conditional):** Depending on the availability and the time to result, multi-gene panel testing or next-generation sequencing can be the first step to further differentiate the underlying pathophysiology in patients with HPA or to confirm BH_4_Ds in patients with a suspicious clinical presentation. The gene panel should include the *QDPR, GCH1, PTS PCBD1, SPR, PAH* and *DNAJC1* genes. If results of genetic testing are unremarkable, consider other known neurotransmitter disorders (e.g. tyrosine hydroxylase deficiency, aromatic l-amino acid decarboxylase deficiency), especially in the case of non-HPA.

### Concluding statements regarding key diagnostic tests

**R#17 (strong):** There are 5 core diagnostic keys for identifying BH_4_D (see Fig. 2 Diagnostic flowchart):

- Elevated Phe levels in NBS or selective diagnostic work-up in patients with AR-GTPCHD, PTPSD, DHPRD or PCDD.
- Abnormal levels of biopterin, neopterin, primapterin and/or sepiapterin in urine and DBS.
- In DHPRD: decreased DHPR enzyme activity in DBS.
- Low CSF levels of 5-HIAA, HVA in combination with altered levels of CSF pterins and/or high sepiapterin in CSF.
- Confirmation of pathogenic variants in the *GCH1, PTS, SRP, QDPR* and *PCBD1* genes.

### Other diagnostic tests: blood prolactin

Dopamine acts as an inhibitor of prolactin secretion. Therefore, prolactin in blood can be elevated in dopamine biosynthesis disorders [17]. Elevated prolactin was found in 22 PTPSD [25, 70, 71], and 3 DHPRD cases [72]. Prolactin was found normal in AD-GTPCHD subjects [73]. In SRD, no reports on elevated prolactin levels were available [21, 23, 74]. For AR-GTPCHD and PCDD, no literature evidence was available.

There are further notable reasons for increased prolactin levels in blood such as physiological or pathological endocrine conditions, hypothalamus and pituitary disorders, systemic disorders, infections, drug related changes, and post-ictal status [75, 76].

Measurement of prolactin in blood is an inexpensive laboratory test that is available worldwide.

**R#18 (research):** Prolactin measurement can be part of the diagnostic work-up for suspected dopamine deficiency, but it has low sensitivity and specificity. Recommendation for further research on prolactin levels at diagnosis and during drug treatment.

### Other diagnostic tests: serotonin (whole blood)

Whole blood serotonin was reported to be low in only 5 cases of SRD [21, 77]. In all other BH_4_Ds, no literature evidence was available. Due to the very limited number of patients, it is not possible to draw conclusions about the diagnostic accuracy of this test.

**R#19 (research):** The role of serotonin measurement in diagnosis and treatment monitoring should be evaluated in further research.

### Other diagnostic tests: BH_4_ loading test

Determination of BH_4_-responsiveness in patients with HPA can be done by oral BH_4_ loading test. The test was initially also used to discriminate between patients with elevated phenylalanine levels due to PAH deficiency and patients with elevated Phe levels due to BH_4_D [78]. At present, there is no uniform test procedure available and test protocols vary considerably from short duration (8 h) to extended duration (48 to 78 h) with repeated BH_4_ administration [79, 80]. Comparably, BH_4_ doses used vary from 2.5 mg to 20 mg/kg BW and higher.

A BH_4_ loading test was performed in 7 studies with > 15 AR-GTPCHD patients, 33 studies with 443 PTPSD patients, in 22 studies with 161 DHPRD patients, and 7 studies with > 12 PCDD patients. All studies have a low or very low level of evidence according to GRADE. There is no literature evidence available for AD-GTPCH and SRD. Regarding the effect of BH_4_ on Phe levels, studies are conclusive, documenting a significant decrease in Phe concentration within the first 8–12 h following BH_4_ load in AR-GTPCHD, PTPSD and PCDD. In contrast, patients with DHPRD show a less prominent Phe reduction during the same time period [3, 81, 82].

Sample collection for the BH_4_ test is minimally invasive. The test, however, requires blood sampling over 8-12 h and placement of a nasogastric tube for patients who refuse to take BH_4_ by mouth.

**R#20 (Conditional):** The oral BH_4_ loading test can be considered in patients with HPA for the assessment of BH_4_-responsiveness.

**R#21 (GPP):** Test procedures for measuring BH_4_ responsiveness can follow local recommendations for HPA patients. The procedure usually consists of baseline assessment of Phe concentration in blood at times − 24 h, − 12 h, and 0 h (=basal measurement). This is followed by the oral administration of 20 mg/kg BW of sapropterin dihydrochloride once daily, taken with a regular meal on two consecutive days. Phe concentration in DBS should be tested every 8 h for 72 h after exposure.

### Other diagnostic tests: Phe loading test

Oral Phe loading has been used additionally for the differential diagnosis of dystonia and BH_4_Ds [83–85]. During the test, hepatic phenylalanine hydroxylase is stressed by an oral Phe intake. In the setting of partial BH_4_ deficiency, conversion of Phe to Tyr is compromised, resulting in an elevated Phe/Tyr ratio for up to 6 h. In addition, the physiological stimulation of BH_4_ biosynthesis via GTPCH feedback regulatory protein (GFRP) by Phe is absent, and biopterin concentrations remain low after Phe loading [86].

The available literature on Phe loading tests in patients with BH_4_Ds is composed of 38 studies with > 31 AR-GTPCHD patients, 13 studies with > 100 AD-GTPCHD patients, one study on one PTPSD patient, 4 studies with 4 DHPRD cases and 4 studies with > 50 SRD patients. While all studies constitute a low or very low level of evidence, the conclusions that can be drawn are consistent: Plasma Phe concentrations are elevated and tyrosine remains unchanged resulting in an increased Phe/Tyr ratio. Further discrimination from heterozygous PKU patients becomes possible by adding the analysis of biopterin in plasma or DBS. The use of specific paediatric cut-off values improves test sensitivity and specificity [86]. However, test results do not correlate with clinical disease severity [87].

Phe loading is a time-consuming procedure and requires blood sampling over 4-8 h. In uncooperative patients, gastric tube placement may be required. The Phe loading test should not be performed concurrently with administration of BH_4_ [88].

**R#22 (Conditional):** The oral phenylalanine loading test can be considered when there is clinical suspicion for AR−/AD-GTPCHD and SRD, when first line diagnostic tests (CSF biogenic amines and pterins or genetic studies) are not available.

### Other diagnostic tests: L-Dopa loading test

A temporary therapeutic L-Dopa trial has been historically commonly used in children or adults with unexplained early-onset dystonia. However, there is a paucity of knowledge available about the sensitivity and specificity of this method. The present need for this test has in addition been questioned given the increasing availability of advanced biochemical, radiological and molecular genetic investigations [89].

**R#23 (GPP):** The diagnostic L-Dopa trial should be limited to children with features suggestive of dopa-responsive dystonia such as lower limb dystonia with diurnal variation and absent HPA. Trial outcome should be monitored by careful clinical assessment including thorough (video supported) documentation of motor dysfunction, autonomic dysfunction, and psychiatric symptoms.

### Other diagnostic test: lumbar puncture (amino acids in CSF)

Only 8 studies with 25 patients (22 cases of AR-GTPCHD, 1 case of DHPRD and 2 cases of SRD) report results of amino acids analysis in CSF. No data is available for AD-GTPCHD, PTPSD, and PCDD. Phenylalanine was normal to high in AR-GTPCHD and high in DHPRD [51]. PCDD and SRD had normal Phe [23, 51, 90, 91].

The measurement of amino acids in CSF is available in many laboratories, mostly located in first world countries.

**R#24 (research)**: Amino acids in CSF are not required for diagnosing BH_4_Ds. For a better understanding of the pathophysiological role of elevated Phe levels in BH_4_Ds under treatment, we recommend the measurement of Phe in CSF for future research studies.

### Other diagnostic test: lumbar puncture (other metabolites in CSF)

Low nitrite/nitrate levels in CSF were reported in 12 cases of PTPSD, 9 cases of DHPRD, and in 1 case of SRD [92]. Low 3-Methoxy-4-hydroxyphenylglycol (MHPG) was found in 5 DHPRD [93, 94] and in 4 SRD [95–97] cases. Low concentrations of 3-Methoxytyramine (3-MT), 3,5-Dihydroxyphenylglycine (DHPG), and 3,4-Dihydroxyphenylacetic acid (DOPAC) were observed in 1 DHPRD case [98]. Noradrenaline/epinephrine in CSF was normal in 1 DHPRD [99] and 1 PCDD patient [100]. Dopamine in CSF was reported to be normal in 1 PCDD case. 5-HTP in CSF was low in 2 SRD cases and normal in 1 case, and L-Dopa was normal in 1 SRD case [95, 97]. No reports on these parameters were available for AD-GTPCH and AR-GTPCH deficiency.

All of these analyses are available only in specialized laboratories, mostly located in first world countries.

**R#25 (research):** Recommendation for research on quantification of CSF biomarkers (including nitrite, nitrate, MHPG, 3-MT, DOPAC) when lumbar puncture is performed for other clinical reasons.

### Key diagnostic test: enzyme activity measurements

A strong recommendation for the analysis of DHPR enzyme activity in DBS in patients with HPA in NBS and/or in case of clinical suspicion of disorders of BH_4_ deficiency has been given above **(R#11).**

Enzyme activity essays are also available for the other BH_4_D and their results are described for 26 AR-GTPCHD, 23 AD-GTPCHD, 91 PTPSD, 53 SRD cases as well as 7 cases with PCD deficiency. As material source, skin fibroblasts, blood (erythrocytes, lymphocytes), liver tissue, and cerebral frontal lobe tissue were chosen. In AR-GTPCHD, PTPSD and SRD, the enzyme activities showed diagnostically relevant reduced concentrations [24, 41, 101–105]. The residual enzyme activity does not correlate with the subsequent disease course. A clear description of the methods and source of tissues for cases of PCDD is not available.

**R#26 (conditional):** Conditional recommendation against enzyme measurement in all other BH_4_ deficiencies for confirmation of the diagnosis since other less invasive and more sensitive diagnostic options are available**.**

### Diagnosis: brain imaging

Cranial magnetic resonance imaging (cMRI) or computer tomography (cCT) results were reported in more than 100 patients with all variants of BH_4_Ds apart from PCDD. All patients with AD-GTPCHD showed normal results. The highest rate of abnormal brain imaging results was reported in patients with DHPRD (all 8 cMRI abnormal, 22 out of 24 cCT abnormal). Neuroradiological findings included brain atrophy, basal ganglia calcifications, white matter changes, ventricular dilatation, areas of hypodensity, and global demyelinating signs [3, 94, 106–112]. In PTPSD, 13/26 cMRI and 3/5 cCT were normal. Documented neuroradiological findings were widespread delayed myelination, periventricular hyperintensities, brain atrophy, and one case of brain calcifications [3, 47, 113–115]. Very few brain imaging studies showed abnormal results in SRD (5/47 cMRI) and AR-GTPCHD (1/10 MRI) [21, 116]. For other imaging modalities like dopamine transporter (DAT)-scan, Fluorodeoxyglucose Positron-emission tomography (FDG PET), and F-Dopa PET, published data is scarce.

The overall evaluation of all relevant brain imaging changes did not reveal any specific pattern of cMRI abnormalities for BH_4_Ds. Therefore, brain imaging is not required for the diagnosis of BH_4_Ds. However, cMRI is typically performed in the work-up of a patient with movement disorders and/or neurodevelopmental delay in order to exclude other underlying diseases. Furthermore, following standards of good clinical care, neuroimaging is always indicated if there is an unexpected deviation in the clinical course of patient already diagnosed with a BH_4_D.

**R#27 (conditional):** Routine imaging of the brain is not required to diagnose BH_4_Ds.

**R#28 (GPP):** In the work-up of patients with movement disorders and/or neurodevelopmental delay, or in case of an unexpected deviation of the clinical course in patients with BH_4_D, cranial MR imaging should be performed.

### Diagnosis: prenatal diagnosis

If a confirmed diagnosis exists in an index patient, prenatal testing in following pregnancies is possible. The early diagnosis is decisive for prenatally initiated treatment like L-Dopa supplementation, which has been shown to be beneficial in AR-GTPCHD patients [68]. Furthermore, the parents and physicians can prepare for adequate peri- and postnatal management. The method of choice is mutation analysis in chorionic villus samples or in amniotic fluid cells [117]. Pterin analysis in amniotic fluid are also possible but not available as a routine diagnostic procedure [118].

**R#29 (strong):** Molecular genetic analysis is the preferred prenatal testing method for all BH_4_Ds.

## Treatment

### First-line treatment

The long-term neurodevelopmental outcome of BH_4_D patients is strongly influenced by the early initiation of effective treatment [3], therefore therapy must not be delayed. Based on evaluation of the literature, evidence exists for (maintenance) drug therapy, including dosage and side effects, for Phe-reduced diet, sapropterin dihydrochloride, L-Dopa with peripheral DC inhibitor (carbidopa or benserazide), 5-HTP, folinic acid, dopamine agonists (DA), selective monoamine oxidase (MAO) inhibitors (MAO-I), anticholinergic agents, catechol-O-methyl transferase (COMT) inhibitors, selective serotonin reuptake inhibitors (SSRIs), benzodiazepines, melatonin, and botulinum toxin injections. For L-Dopa without DC inhibitor, psychiatric therapy, baclofen, and surgery treatment there was insufficient literature available to inform any recommendations.

Note: For all treatment options, the total body of evidence in the literature was rated as low or very low. Multiple studies referred to the same data basis within the BIODEF database. Positive, neutral, and negative (“side effects”) treatment effects are summarized in the following sections. Detailed dose recommendations are given in Table 4.

**Table 4:** Recommended drugs and doses for BH_4_ disorders

	Disorder	Starting dose	Doses	Target dose	Maximum dose	Management suggestion	Comment
**First line treatment**							
**Phe-reduced diet**	All BH_4_D with HPA					Titrate Phe restriction according to Phe levels in DBS or plasma	Follow PKU national treatment recommendations Use either Phe reduced diet or Sapropterin dihydrochloride to control Phe levels
**Sapropterin dihydrochloride**	All BH_4_D with HPA apart from DHPRD	2-5 mg/kg BW/day	Divided in 1–3 doses/ day	5–10 mg/kg BW/day	20 mg/kg BW/day	Titrate dose according to Phe levels in DBS or plasma	Follow PKU national treatment recommendations Use either Phe reduced diet or Sapropterin dihydrochloride to control Phe levels
**L-Dopa/DC inhibitor (carbidopa/benserazide) 4:1**	All BH_4_D apart from PCDD	0.5 mg–1 mg/kg BW/day Dose recommendation relates to L-Dopa component!	Divided in 2–6 doses/ day	AD-GTPCHD: 3–7 mg/kg BW/day All other BH_4_D: 10 mg/kg BW/day or maximally tolerated dosage Dose recommendation relates to L-Dopa component!	Depending on clinical symptoms. Some patients need more than 10 mg/kg BW/day for resolving clinical symptoms	Increase 0.5–1 mg/kg BW/day per week Follow BW adaption until the BW of 40 kg. After 40 kg adjust depending on clinical symptoms Consider analysis of CSF HVA for dose adjustment	In young infants at least as many dosages as meals would be ideal (usually 5–6 /day)
**5-Hydroxytryptophan (5-HTP)**	All BH_4_D apart from AD-GTPCHD and PCDD	1–2 mg/kg BW/day	Divided in 3–6 doses/day	Published target dose recommendations are highly variable 5-HTP doses are usually lower than L-Dopa doses		Titrate slowly (1–2 mg/kg BW/day per week) depending on clinical picture and side effects Consider analysis of CSF 5HIAA for dose finding	5-HTP should follow L-Dopa/DCI treatment initiation Always in combination with a peripheral decarboxylase inhibitor (for example by simultaneous application with L-Dopa/DC inhibitor)
**Folinic acid**	In DHPRD and all BH_4_D with low 5-MTHF in CSF		Divided in 1–2 doses/day	10–20 mg/day		No titration needed Consider analysis of CSF 5MTHF for dose finding	
**Second line treatment**							
**Pramipexole**^**a**^ (Dopamine agonist)	All BH_4_D apart from PCDD	3.5–7 μg/kg/BW/day (base) 5–10 μg/kgBW/day (salt) Note: Distinction in salt and base content! (see product insert)	Divided in 3 equal doses/day	Titrate to clinical Symptoms	75 μg/kg BW/day (3.3 mg/d base / 4 mg/d salt)	Increase every 7 days by 5 μg/kg BW/d	
**Bromocriptine**^**a**^ (Dopamine agonist)	All BH_4_D apart from PCDD	0.1 mg/kg BW/day	Divided in 2–3 doses/day	Titrate to clinical Symptoms	0.5 mg/kg/d (or 30 mg/d)	Increase every 7 days by 0.1 mg/kg BW/d	
**Rotigotine**^**a**^ (transdermal dopamine agonist)	All BH_4_D apart from PCDD	2 mg/day		Titrate to clinical Symptoms	8 mg/day	Increase weekly by 1 mg	Children > 12 years Exchange patch every 24 h
**Selegiline**^**a**^ (MAO B inhibitor)	All BH_4_D apart from PCDD	0.1 mg/kg BW/day ATTENTION: orally disintegrating preparation needs much less dosage because of missing first-pass effect in the liver	Divided in 2 (−3) doses/day	Titrate to clinical Symptoms	0.3 mg/kg/d (or 10 mg/d)	Increase every 2 weeks by 0.1 mg/kg BW/d	Can cause sleep disturbances – morning and afternoon or lunchtime dosage is possible ATTENTION: orally disintegrating preparation needs much less dosage because the first-pass effect of the liver is avoided
**Third line treatment**							
**Trihexyphenidyl**^**a**^ (Anticholinergic drugs)	All BH_4_D apart from PCDD	< 15 kg: start 0.5–1 mg/day > 15 kg: start 2 mg/day	< 15 kg: in 1 dose > 15 kg: in 2 doses	Effective dose highly variable (6–60 mg) Titrate to clinical Symptoms	Maximum dose: < 15 kg BW 30 mg/day > 15 kg BW 60 mg/d	Increase every 7 days by 1–2 mg/d in 2–4 doses/d	Consider side effects: like dry mouth, dry eyes, blurred vision (mydriasis), urine retention, constipation.
**Entacapone**^**a**^ (COMT inhibitor)	All BH_4_D apart from PCDD	200 mg (adult)			Up to 2.000 mg		In many countries licensed only for adults. Comedication with L-Dopa/DC inhibitor Consider reduction of concomitant L-Dopa supplementation (10–30%)
**Sertaline**^**a**^ (SSRI)	All BH_4_D apart from PCDD	6–12 years: 25 mg/day in 1 dose > 12 years: 50 mg/day in 1 dose	6–12 years: in 1 dose > 12 years: in 1 dose	Children 50 mg/day	50 mg/day < 12 years 200 mg/day > 12 years	6–12 years: increase after 7 days to 50 mg/day in 1 dose > 12 years 50 mg/day in 1 dose	Don’t stop treatment suddenly Note: Elevated risk of serotonin syndrome (SS) or malignant neuroleptic syndrome (MNS) when used with drugs impacting serotonergic pathway (e.g. 5-HTP, MAO inhibitors)
**Melatonin**^**a**^	All BH_4_D apart from PCDD	0.01–0.03 mg/kg/day			5–8 mg/day		Slow release preparation for sleep-maintenance insomnia available in some countries

Please note: The doses given are in a range typically used and have been published. In individual patients, some adjustment may be necessary depending on symptom response and side effects

^a^The evaluated literature did not provide BH_4_D specific treatment dose recommendations for this drug. The listed doses, therefore, indicate treatment recommendations from Summary of Product Characteristics (SmPC) or neurotransmitter related publications (e.g. [119])

*Abbreviations*: *5-HIAA* 5-hydroxyindoleacetic acid, *5-HTP* 5-hydroxytryptophan, *5-MTHF* 5- methyltetrahydrofolate, *HVA* Homovanillic acid, *AD-GTPCHD* Autosomal-dominant GTPCHD: guanosine triphosphate cyclohydrolase I deficiency, *BH*_*4*_*D* Tetrahydrobiopterin deficiency, *BW* Body weight, *COMT* Catechol-O-methyl transferase, *CSF* Cerebrospinal fluid, *DBS* Dry blood spot, *DC* Decarboxylase, *DCI* Decarboxylase inhibitor, *DHPRD* Dihydropterin reductase deficiency, *L-Dopa* L-3,4-dihydroxyphenylalanine, *MAO B* Monoamine oxidase B, *PCDD* Pterin-4-alpha-carbinolamine dehydratase deficiency, *Phe* Phenylalanine, *PKU* Phenylketonuria, *SSRI* Selective serotonin reuptake inhibitor

### Dietary treatment

#### Phe-reduced diet

The harmful effect of Phe accumulation in blood and brain is best witnessed in untreated patients with PKU who develop irreversible neurological impairment and psychiatric symptoms if exposed to HPA [5]. Although the precise pathogenesis of brain dysfunction is still unclear, there is strong rationale for reducing HPA and maintaining satisfactory low Phe levels in all BH_4_Ds with HPA [120]. The two available strategies to treat HPA are a Phe-reduced diet or sapropterin dihydrochloride supplementation.

Phe-reduced diet was used in 5 AR-GTPCHD patients in 5 studies, 103 PTPSD patients in 25 studies, 115 DHPRD patients in 40 studies, and 29 PCDD patients in 5 studies. Use of the Phe-reduced diet was not reported in any patient with SRD or AD-GTPCHD.

There is no precise data available regarding the daily Phe tolerance in patients with BH_4_D and no precise data on the number of patients who, after being originally treated solely with a Phe-reduced diet, later switched to sapropterin dihydrochloride supplementation or a combined Phe-reduced diet plus sapropterin dihydrochloride treatment.

Clear positive effect of Phe-reduced diet on lowering Phe levels was documented in the vast majority of patients. However, as HPA is only one of the pathophysiological mechanisms in BH_4_Ds, Phe-reduced diet as monotherapy in AR-GTPCHD, PTPSD, and DHPRD failed to improve symptoms such as hypotonia, hypertonia, developmental delay, dystonia, other involuntary movements, sleep and mood disturbances or epileptic seizures. In contrast, there are 2 reports of PCDD patients with muscular hypo/−or hypertonia and motor developmental delay who showed improvement following treatment with a Phe-reduced diet only [53].

In the reviewed BH_4_Ds literature, no negative effects of Phe-reduced diet have been documented. However, unnecessary dietary restrictions should be avoided, and daily Phe intake and tolerance closely monitored to optimize the maximal natural protein intake. Symptoms of Phe deficiency may include anorexia, listlessness, alopecia, perineal rash, poor growth or even death [5].

Note: As Phe levels in PCDD have nearly always been reported to be only mildly elevated, relaxation and discontinuation of Phe-reduced diet and/or sapropterin dihydrochloride supplementation can be attempted after the first year of life under careful monitoring of Phe levels.

**R#30 (strong):** Phe control should be applied in BH_4_Ds with HPA (AR-GTPCHD, PTPSD, DHPRD and PCDD). Phe control may be achieved by Phe-reduced diet or sapropterin dihydrochloride supplementation (see below). Phe levels should be regularly monitored in DBS or blood. Target ranges should be determined by local recommendations for the dietary treatment of PKU.

**R#31 (strong):** Phe-reduced diet should not be used in BH_4_Ds without HPA (AD-GTPCHD and SRD).

**R#32 (strong):** Phe-reduced diet should not be used in monotherapy for the treatment of neurological symptoms in BH_4_Ds.

#### Drug treatment

##### Sapropterin dihydrochloride

The rationale for supplementation of sapropterin dihydrochloride, a synthetic tetrahydrobiopterin analogue, is based upon the defective biosynthesis or recycling of this essential cofactor for the aromatic L-amino acid hydroxylases in all types of BH_4_Ds. The main effect of sapropterin dihydrochloride supplementation lies within its marked impact on the control of peripheral Phe levels. Animal studies have demonstrated rather poor penetration of peripherally administered sapropterin dihydrochloride across the blood-brain barrier and an increase of total biopterin only after supplementation with doses not applicable in clinical practice [121, 122]. Data concerning BH_4_ uptake into the human brain, and correction of dopamine and serotonin metabolism following sapropterin dihydrochloride administration, are very limited too [122]. Although sapropterin dihydrochloride is more expensive than a Phe-reduced diet and is still unavailable in some (European) countries, it enables a markedly higher natural protein intake and offers a much more convenient way to treat HPA. Pharmacokinetic studies indicate a mean elimination time of around 4–7 h depending on the population studied. It can be administered in one daily quantity and the target Phe concentrations should follow the national recommendation for the treatment of PKU.

The literature search identified approximately 40 AR-GTPCHD patients from 16 studies, 397 PTPSD patients from 41 studies, 194 DHPRD patients from 19 studies (see note below), 29 PCDD patients from 10 studies, and 10 SRD patients from 9 studies treated with sapropterin dihydrochloride, while no AD-GTPCHD patient on sapropterin dihydrochloride was identified.

Evaluation of specific treatment effects of sapropterin dihydrochloride was hampered by the use of co-treatment (L-DOPA/DC inhibitor, 5-HTP) in almost all patients, except in PCDD. The assessment of the effects of sapropterin dihydrochloride may therefore be biased.

Similar to a Phe-reduced diet, if co-administered with neurotransmitter precursors, a sapropterin dihydrochloride treatment consistently improved almost all clinical symptoms in PTPSD, DHPRD (see comment below), and AR-GTPCHD, including movement disorders (dystonia, oculogyric crises, choreoathetosis, tremor, hypotonia, hypertonia, rigidity), epileptic seizures, sleep problems, gastrointestinal disturbances (hypersalivation, swallowing difficulties), anthropometric parameters, developmental delay, and behavioural abnormalities. In addition, biochemical markers such as the concentration of CSF neurotransmitter metabolites were positively influenced. In SRD patients, however, no clear clinical benefit was reported [21, 96].

Experience of sapropterin dihydrochloride monotherapy is mainly limited to mild forms of PTPSD with minimal or even absent clinical symptoms, and normal levels of dopamine and serotonin metabolites in CSF [44]. However, such patients need to be closely monitored as evolution from a mild into a severe phenotype can occur, requiring the full treatment regimen with dopamine and serotonin precursors [123]. A handful of patients experienced improvement in developmental impairment, hypotonia, swallowing difficulties, hypersalivation, drowsiness or epileptic seizures when on sapropterin dihydrochloride monotherapy.

If used as monotherapy, sapropterin dihydrochloride has been reported in several cases to fail to improve or prevent intellectual disability, movement disorders, seizures or sleep problems, and it also did not affect the levels of CSF neurotransmitter metabolites [22, 25, 44].

No negative effects related to sapropterin dihydrochloride administration have been reported in the literature reviewed. According to the official drug information, patients may commonly (≥1/10) or very commonly (≥1/100 to < 1/10) experience headaches, rhinorrhoea, pharyngolaryngeal pain, nasal congestion, cough, diarrhoea, abdominal pain, dyspepsia or nausea.

Note: Based on the hypothesis that sapropterin dihydrochloride supplementation may lead to increased 7,8-dihydrobiopterin (BH_2_) production and a decreased BH_4_/BH_2_ ratio resulting in aggravation of disease severity by inhibiting the aromatic L-amino acid hydroxylases or by increasing nitric oxide (NO) uncoupling and oxidative stress, this treatment approach is currently controversial in DHPRD [124, 125]. However, literature evidence for these potential harmful effects is scarce and based on cell experiments only [124]. In contrast, there are 194 patients with DHPRD (15 studies) received BH_4_ supplementation published who did not show any clinical or biochemical adverse effect that could be directly related to BH_4_ supplementation. Furthermore, there is a suggestion that restoration of the BH_4_ pool with BH_4_ supplementation may have a protective effect [125]. Therefore, there is no reliable justification to withhold this therapeutic intervention from patients with DHPRD.

**R#33 (strong):** Phe control should be applied in BH_4_Ds with HPA (AR-GTPCHD, PTPSD, DHPRD and PCDD). Phe control is possible through a Phe-reduced diet (see above) or through administration of sapropterin dihydrochloride. Sapropterin dihydrochloride is the treatment of choice in AR-GTPCHD, PTPSD, and PCDD. It should be administered once daily and doses should be titrated according to Phe levels. Phe levels should be controlled in DBS or blood, and target ranges should follow local recommendations for the dietary treatment of PKU. In PCDD, discontinuation of sapropterin dihydrochloride supplementation can be attempted after the first year of life under careful Phe level monitoring.

**R#34 (conditional):** In DHPRD, Phe-reduced diet and not the supplementation of sapropterin hydrochloride is nowadays considered the method of choice for the control of HPA. Since the available evidence against the use of sapropterin dihydrochloride is scarce, sapropterin dihydrochloride can be considered in DHPRD patients. Phe levels should be controlled in DBS or blood, and target ranges should follow local recommendations for the dietary treatment of phenylketonuria.

**R#35 (research):** To better understand the pathophysiological mechanism and metabolic consequences of sapropterin dihydrochloride treatment in DHPRD and also the effect of sapropterin dihydrochloride monotherapy, we recommend further research on these topics.

##### L-Dopa with or without carbidopa/benserazide

BH_4_Ds result in significantly reduced dopamine availability in the CNS. L-Dopa, a dopamine precursor that is converted to dopamine by the aromatic L-amino acid decarboxylase (AADC) enzyme, has been widely used for numerous indications in order to restore dopamine homoeostasis. The addition of carbidopa or benserazide, a peripheral DC inhibitor, blocks the peripheral decarboxylation of L-Dopa. This results in increased L-Dopa concentrations at the blood-brain barrier and also in reduced peripheral L-Dopa side effects.

The effect of L-Dopa/carbidopa was described in 197 AD-GTPCHD patients in 32 studies, in 45 AR-GTPCHD patients in 18 studies, in 540 PTPSD cases in 36 studies, in 249 DHPRD patients in 45 studies, in 49 SRD patients in 25 studies, and in one 1 PCDD patient.

The effect of L-Dopa/benserazide was documented in 11 AD-GTPCHD patients in 6 studies, in 11 PTPSD patients in 2 studies and in 4 SRD patients in 4 studies. For L-Dopa/benserazide treatment in AR-GTPCHD, DHPRD, and PCDD, no literature evidence is available.

It is not possible to judge the effect of L-Dopa without a DC inhibitor since for 162 AD-GTPCHD patients in 32 studies, 1 AR-GTPCHD patient in 1 study, 47 PTPSD patients in 4 studies, 21 DHPRD patients in 7 studies, and 1 SRD patient, it was not clearly stated whether L-Dopa was used alone or in combination with a DC inhibitor. For PCDD, no literature evidence is available on the use of L-Dopa without a DC inhibitor.

Note: Almost invariably, L-Dopa/DC inhibitor treatment was initiated simultaneously with 5-hydroxytryptophan treatment (apart from AD-GTPCHD). Evaluating the impact of these drugs separately may therefore be problematic and biased. Furthermore, the patients were concurrently treated with sapropterin hydrochloride and on a Phe-reduced diet.

L-Dopa/DC inhibitor treatment in BH_4_D patients was found to improve most disease-related symptoms in the majority of studies. Positive treatment response correlated inversely with the age at treatment initiation [3, 82], however, treatment non-responders were reported too.

The positive effects of L-Dopa/DC inhibitor treatment in BH_4_Ds were observed on almost all clinical endpoints. Improvements in motor development, cognitive functions, muscle tone abnormalities (hypotonia, poor head control, hypertonia) and epileptic seizures were reported in the largest proportion of patients. A positive effect could also be observed on dystonia, including oculogyric crises, dyskinesias and other movement disorders, parkinsonism, epileptic seizures, autonomic dysregulation (temperature instability), gastrointestinal disturbances (hypersalivation, swallowing difficulties), anthropometric parameters (failure to thrive, growth retardation, microcephaly), sleep problems, behavioural and psychiatric disorders or delayed speech development. Additionally, a positive effect of L-Dopa/DC inhibitor treatment was reported on the level of various biochemical markers, including urinary pterins, CSF neurotransmitter metabolites and prolactin.

In some patients, however, regardless of the BH_4_D type, even combined treatment with L-Dopa/DC inhibitor failed to improve either disease symptoms or biomarker results.

Negative effects reported in BH_4_D patients receiving L-Dopa/DC inhibitor treatment correspond to the adverse effects generally observed with L-Dopa treatment. The negative motor effects manifested mainly as dyskinesia and as motor fluctuations with on/off phenomenon. Other movement disorders (tremor, chorea, myoclonic jerks) were observed less frequently. Non-motor side effects of L-Dopa/DC inhibitor described included behavioural and psychiatric symptoms (anxiety, delusions, impulsivity, irritability, hyperactivity, mood fluctuations or panic attacks), sleep disturbances, gastrointestinal problems (nausea, vomiting, diarrhoea) and headaches.

The most commonly used ratio of L-Dopa to DC inhibitor is 4:1. The available literature doesn’t allow an evaluation of whether the 4:1 preparation has a superior effect compared to the 10:1 preparation. According to published drug information, there is no known upper dose limit for DC inhibitor or any specific side effects described. In contrast, side effects of L-Dopa in the context of DC inhibitor underdosing clearly justify sufficient dosing.

From a pharmaceutical perspective, it is important to mention that benserazide is unstable in the air. During compounding, the substance oxidizes and can therefore become ineffective. Carbidopa, on the other hand, is relatively stable. It can also be readily compounded into a formulation suitable for children. Theoretically, drug forms for the preparation of suspensions are available. However, since there is no uniform distribution in the suspension due to the undissolved particles, it is not recommended to divide a suspension. If a suspension is used, it should be administered immediately after production.

**R#36 (strong):** L-Dopa should always be given in combination with a DC inhibitor (4:1 ratio) and should be the first line of treatment in AD-GTPCHD, AR-GTPCHD, DHPRD, PTPSD, and SRD.

**R#37 (strong):** The L-Dopa/DC inhibitor starting dose should be low, distributed in several daily dosages and slowly titrated depending on the clinical symptoms. In case of side effects, the timing and dosing of medication may be adjusted individually. Referring to the normal range of dopamine metabolite values in CSF, which is highest during the neonatal and infantile period, the target treatment dose in all infants (below 40 kg body weight) with BH_4_D **(apart from AD-GTPCHD)** is 10 mg/kg BW/d (if clinically tolerated). Some patients require higher doses. In AD-GTPCHD, most patients obtain complete symptom control with lower doses of L-Dopa /DC inhibitor.

##### 5-Hydroxytryptophan

Reduced bioavailability of serotonin in the CNS in BH_4_Ds results from impaired conversion of tryptophan to 5-HTP by tryptophan hydroxylase 2 (TPH2), for which BH_4_ is an essential cofactor. The subsequent conversion of 5-HTP to serotonin is carried out by the AADC enzyme, which is unaffected in BH_4_Ds. This forms the pathophysiological rationale for the supplementation of 5-HTP in BH_4_Ds with a potential to correct the neurotransmitter imbalance.

5-HTP was used in 41 AR-GTPCHD cases in 12 studies, in 4 patients with AD-GTPCHD in 1 study, in 542 PTPSD patients in 41 studies, in 93 DHPRD in 49 studies, 14 SRD patients in 19 studies, and in 1 PCDD patient.

For all patients, except for one patient with PCDD, overall clinical improvement on various endpoints was reported.

Note: 5-HTP was used in combination with other medications in all patients. 5-HTP treatment is often initiated simultaneously with L-Dopa/DC inhibitor and/or sapropterin dihydrochloride /Phe-reduced diet, which markedly hampers the assessment of the effects of 5-HTP alone. 5-HTP treatment was started without L-Dopa/DC inhibitor in only in a handful of patients; the reported effects are inconsistent among the studies.

The observed positive effects of 5-HTP (at least in co-administration with L-Dopa/DC inhibitor) include improvement in almost all clinical endpoints including acquisition of developmental milestones, cognition, tone and movement disorders, epileptic seizures, swallowing difficulties and hypersalivation, speech development, attention and behaviour, and mood (depression). Sleep disturbances have been reported to improve with 5-HTP treatment. Due to co-medication, the improvement could, however, only be clearly assigned to 5-HTP supplementation in very few patients. Psychiatric and behavioural problems, other symptoms often associated with serotonin deficiency, were reported to improve in some patients as well: In 4 AD-GTPCHD patients, depression improved on 5-HTP in monotherapy or 5-HTP in combination with serotonin agonists or serotonin reuptake inhibitors [126]. Interestingly, during a 5-HTP shortage lasting for 6 months, no obvious neurologic deterioration could be observed in a cohort of 12 PTPSD patients [127].

As with L-Dopa/DC inhibitor treatment, 5-HTP administration failed to improve symptoms in some patients.

The most common adverse effects of 5-HTP are gastrointestinal problems (nausea, vomiting, diarrhoea, abdominal pain). Indeed, these symptoms necessitated 5-HTP discontinuation in some cases. Irritability, choreoathetoid, dyskinetic or myoclonic movement disorders, and sweating were observed, too. Given its co-administration with L-Dopa/DC inhibitor, numerous other adverse effects were observed, but from a pathophysiologic standpoint, these are more likely to be related entirely or at least partially to L-Dopa rather than to 5-HTP treatment.

**R#38 (strong):** From a biochemical standpoint, 5-HTP is considered a first line treatment in BH_4_Ds. In patients with DHPRD, PTPSD, and SRD, benefits clearly outweigh adverse effects, leading to a strong recommendation for the use of 5-HTP in these disorders. For PCDD and AD-GTPCHD, no recommendation can be given due to lack of evidence.

**R#39 (conditional):** For AR-GTPCHD, desirable consequences probably outweigh undesirable consequences, thus forming a conditional recommendation for the use of 5-HTP in this disorder.

**R#40 (strong):** 5-HTP should follow initiation of the L-Dopa/DC inhibitor treatment. There is no clear evidence for a starting dose; however, it should be lower than the L-Dopa dose (e.g. Table 4). It should not be changed at the same time as L-Dopa to clearly distinguish clinical effects. Start with a low dose and titrate slowly as dictated by clinical symptoms. Use a peripheral decarboxylase inhibitor (e.g. by administering at the same time as L-Dopa/DC inhibitor) to reduce (gastrointestinal) side effects.

##### Folinic acid

Cerebral folate deficiency may occur in BH_4_Ds, most prominently in DHPRD. However, there is risk of development of cerebral folate depletion in the other BH_4_Ds too, as long-term administration of L-Dopa in high doses can result in reduced availability of these methyl groups due to the methylation of L-Dopa to 3-*O*-methydopa (3-OMD) [51].

The therapeutic use of folinic acid in BH_4_Ds is reported in more than 14 AR-GTPCHD cases in 3 studies, in approximately 40 PTPSD patients in 2 studies, in 262 DHPRD cases in 37 studies, and in 1 SRD patient. There is no literature available for the use of folinic acid in PCDD and AD-GTPCHD.

Assessment of the clinical efficacy of folinic acid supplementation is substantially influenced by the use of various co-medications in the vast majority of patients or by the lack of data on the clinical course following the introduction of folinic acid.

The positive effects of folinic acid (in combination with other medications) included improvement in motor and cognitive function in movement disorders or epileptic seizures. The rare reports on the change of a patient’s clinical status after introducing folinic acid claimed improvement in overall condition, in seizure control and neurologic status, and in the CSF neurotransmitter profile [63, 128, 129]. In the only DHPRD patient treated solely with folinic acid monotherapy (apart from Phe-reduced diet), improvement of tremor, drowsiness, hypersalivation, and in the frequency of myoclonic seizures was noticed [130].

In some patients, the addition of folinic acid did not change the clinical status or improve the CSF neurotransmitter profile [24, 125].

In 2 patients, adverse effects consisting of vomiting, irritability, and changes in sleep pattern were observed; however, these patients were treated concurrently with other medications. Otherwise, there are no reports of negative effects that could be related to the addition of folinic acid supplementation to the treatment regimen.

It is important to note that folic acid, a non-naturally occurring form of folate used to fortify food, is contraindicated in this condition, as it competitively binds to the folate receptor alpha (FRα), resulting in reduced 5-MTHF transport into the brain [61].

**R#41 (strong):** Folinic acid supplementation should be used in patients with DHPRD. Note: Cerebral folate deficiency may even be aggravated by the administration of folic acid!

**R#42 (conditional):** Folinic acid supplementation should be considered in any patient with BH_4_D found to have low 5-MTHF concentration in CSF.

### Second-line treatment

#### Drug treatment

##### Dopamine agonists

Dopamine agonists (DA) exert their function by direct postsynaptic activation of dopamine receptors. Ergot-derived DAs that have a strong serotonergic (5HT2b) receptor interaction (cabergoline and pergolide) are associated with cardiac valvulopathy and other fibrotic adverse events, and have been removed from the market in many countries. Ergot-derived DA without 5HT2b agonist action (bromocriptine) have an overall lower risk. However, pulmonary, retroperitoneal, and (peri) cardial fibrosis have been described with a dose-effect relationship [131]. Non-ergot DAs (apomorphine, piribedil, pramipexole, ropinirole and rotigotine) seem to possess a very low and statistically insignificant risk of fibrotic complications and are preferred in clinical practice [131]. Potential benefits of DAs are related to their longer bioavailability in the synaptic cleft leading to equalised L-Dopa/DC inhibitor stimulation of dopaminergic terminals in the striatum [132].

The use of DA in BH_4_Ds as complementary drug treatment was documented in 12 AD-GTPCH patients, 5 PTPSD and 5 DHPRD patients, and in 8 patients with SRD. No AR-GTPCHD or PCDD patient receiving DA has been reported.

The most commonly used DA was pramipexole (16 patients) followed by bromocriptine (10 patients) and cabergoline (5 patients). Reports on the use of other DA are very scarce and limited to AD-GTPCHD.

Among BH_4_D patients, DAs were most commonly used concomitantly with L-Dopa/DC inhibitor, 5-HTP, sapropterin dihydrochloride, Phe-reduced diet or folinic acid treatment, rarely in monotherapy or in combination with a MAO inhibitor. In the majority of patients, the use of DAs allowed for a significant reduction of L-Dopa/DC inhibitor doses, less frequent daily administrations and an improvement of the residual motor symptoms (if specified, mainly parkinsonian symptoms such as tremor, bradykinesia, hypomimia, dysarthria etc.). Furthermore, DAs were reported to beneficially affect L-Dopa/DC inhibitor adverse effects, namely L-Dopa induced dyskinesia and mood swings [133–139].

Reported DA side effects comprise mainly behavioural/psychiatric disorders; impulse control disorders, including pathological gambling, compulsive buying, and hypersexuality, were reported almost exclusively under pramipexole treatment [136]. Symptoms were dose- dependent and subsided after adequate treatment adjustment. In a few patients, worsening of motor symptoms (dystonia, dyskinesias), weight loss or unspecified negative events led to the discontinuation of DA treatment [32, 137]. No fibrotic complications have been described.

**R#43 (conditional):** Dopamine agonists can be considered as second line treatment in all BH_4_Ds (apart from PCDD) in combination with first line treatment options if residual symptoms persist despite L-Dopa/DC inhibitor treatment or if dose-limiting L-Dopa/DC inhibitor- associated adverse events occur. Non-ergot derived DAs (pramipexole, ropinirole, rotigotine) or ergot-derived DA without 5HT2b agonist action (bromocriptine) are preferred.

**R#44 (GPP):** Cardiac screening before and during treatment with bromocriptine (ergot derived DA) is indicated because of the potential risk of cardiac fibrosis.

##### Selective monoamine oxidase (MAO) inhibitors

MAO inhibitors prevent the breakdown of dopamine and serotonin in the synaptic cleft. The effect of selective MAO inhibitors was described in more than 2 AR-GTPCHD cases in 2 studies, in 4 AD-GTPCHD patients in 4 studies, in roughly 19 PTPSD patients in 5 studies, in 8 DHPRD cases in 4 studies, and in more than 7 SRD patients in 7 studies. For PCDD, no evidence is available. All studies describe the effect of selective MAO inhibitors only in combination with L-Dopa/DC inhibitor, dopamine agonists, 5-HTP, sapropterin dihydrochloride, selective serotonin reuptake inhibitors (SSRIs), Phe-reduced diet or folinic acid. Selegiline (*n* = 36 cases) and rasagiline (*n* = 1 case) are the only selective MAO inhibitors used [31]. No studies were found on tranylcypromine or phenelzine. The majority of studies described an improvement in at least one clinical endpoint (e.g. dystonia, fatigability, sleep, motor development or seizure control) or in lowering L-Dopa doses; an effect on motor fluctuations (on-off phenomena) was described, too. Some studies reported an unclear outcome. Side effects of MAO inhibitors alone (diarrhoea or constipation, drowsiness or insomnia, dry mouth) were not reported. One patient with SRD developed dyskinesia after adding SSRI to the treatment regimen [104].

**R#45 (conditional):** MAO inhibitors can be considered as second line treatment in AR-GTPCHD, AD-GTPCHD, PTPSD, DHPRD, and SRD in combination with first line treatment options although little or no evidence is available.

**R#46 (GPP):** The members of the guideline group consider selective MAO inhibitor a treatment option in case of dose-related symptom fluctuations and drug-induced dyskinesia or motor fluctuations. Use should be guided by availability of the drug and experience of the treating physician. The members of the guideline group judged MAO inhibitors to have fewer side effects compared to dopamine agonists.

### Third-line treatment

#### Drug treatment

##### Anticholinergic drugs

Anticholinergic drugs (e.g. trihexyphenidyl) are commonly used to treat movement disorders, especially dystonia and parkinsonism. The current hypothesis is that anticholinergic drugs influence the relative imbalance between dopaminergic and cholinergic pathways, however, the exact mechanism of action is unclear [140]. The effect of anticholinergic drugs in BH_4_Ds was described in 17 patients with AD-GTPCHD in 8 studies and in 4 patients with SRD in 3 studies. For AR-GTPCHD, PTPSD, DHPRD, and PCDD, no published evidence is available.

Trihexyphenidyl (> 15 cases), benztropine (> 2 cases), and methixene (> 1 case) were the anticholinergic drugs used [139]. In AD-GTPCHD, most patients first received L-Dopa/DC inhibitor. Trihexyphenidyl was added due to incomplete control of symptoms on L-Dopa/DC inhibitor alone and/or due to dyskinesia at higher L-Dopa/DC inhibitor doses [141, 142]. In the majority of AD-GTPCHD patients, a moderate to excellent effect on dystonia and tremor was noted; however, not all patients exhibited clinical benefit. For SRD, positive effects of benztropine supplementation are described in two patients without providing further details. Typical anticholinergic side effects (dry mouth, dry eye, blurred vision (pupil dilation), constipation, urinary retention, reduced sweating) were not described in BH_4_Ds.

**R#47 (conditional):** Consider anticholinergic agents as third-line treatment in AD-GTPCHD and, based on the pathophysiological background, also in AR-GTPCHD, SRD, PTPSD, and DHPRD patients in case of incomplete control of symptoms with L-Dopa/DC inhibitor. For PCDD, no recommendation is possible due to lack of evidence.

##### COMT inhitibors

The inhibitors of catechol-O-methyl transferase (COMT) prevent the action of this enzyme, which is involved in the breakdown of catecholamines and, thus, has a direct impact on the pharmacodynamic and pharmacokinetic properties and the behaviour of levodopa. By this mechanism, COMT inhibitors have the potential to increase the availability of catecholamine neurotransmitters in the CNS, particularly dopamine. The indication for this add-on treatment would be to reduce motor fluctuations associated with L-Dopa treatment. The only evidence available in the literature for treatment of BH_4_Ds is for entacapone.

Treatment with entacapone was described in 4 AD-GTPCHD cases in 3 publications, 8 PTPSD cases in 4 studies, in 14 DHPRD patients in 3 studies. For AR-GTPCHD, PCD, and SRD, no literature evidence is available. There are no descriptions of COMT inhibitor monotherapy in the BH_4_D cohort. All patients were treated concurrently with L-Dopa/DC inhibitor, DA, 5-HTP, sapropterin dihydrochloride, Phe-reduced diet or folinic acid.

The assessment of clinical efficacy was yet again problematic due to insufficient data on the clinical course after initiating COMT inhibitors. Clear positive effects could not be obtained for PTPSD and AD-GTPCHD. Decreased prolactin levels were documented after entacapone introduction in DHPRD. No specific side effects commonly related to COMT inhibitors (dyskinesia, hyper−/hypokinesia, gastrointestinal problems with nausea, constipation or diarrhea) were reported among BH_4_D patients. General dose recommendations can be used.

**R#48 (conditional):** The use of COMT inhibitors can be considered as third line treatment in all BH_4_Ds apart from PCDD. The members of the guideline group consider COMT inhibitors as a treatment option in patients suffering from motor fluctuations with L-Dopa/DC inhibitor treatment.

##### Selective serotonin reuptake inhibitors (SSRI)

SSRIs act by decreasing the presynaptic reuptake of serotonin, which leads to its prolonged bioavailability in the synaptic cleft and better postsynaptic receptor occupancy. The rationale for their use in BH_4_Ds is the presence of symptoms attributable to serotonin deficiency such as psychiatric and behavioural disorders and sleep problems.

The use of SSRIs (sertraline, fluoxetine and others) was reported in 1 patient with AR-GTPCHD, 11 patients with AD-GTPCHD in 4 studies and in 4 patients with SRD in 5 studies. For PCD, DHPRD and PTPSD no literature evidence is available.

For all patients with SRD, improvement of disease related symptoms (including improved alertness, sleep time and dystonia) were documented [96, 104]. In 10 out of 11 patients with AD-GTPCHD, depression improved [126, 143]. One patient with AR-GTPCHD experienced worsening of depression [26], and one patient with SRD had akathisia and dyskinesia/myoclonus while treatment concurrently with a MAO inhibitor (selegiline) [104].

**R#49 (conditional):** There is a conditional recommendation for the use of SSRIs in AD-GTPCHD for psychiatric symptoms.

**R#50 (conditional):** Based on the current evidence, no definitive recommendation can be given for the use of SSRIs in AR-GTPCHD, PTPSD, DHPRD, and SRD. Guideline group members consider SSRIs in individual cases as third line treatment with caution of possible side effects if all first- and second-line treatment options have, over an adequate amount of time, shown to be insufficient to control symptoms. For PCDD, no recommendation is possible due to lack of evidence.

**R#51 (GPP): Caution:** According to the guideline group members, the combination of 5-HTP and SSRI treatment, specifically in very high doses, can induce serotonin syndrome!

##### Melatonin

From a pathophysiological perspective, melatonin supplementation for disorders of sleep induction is reasonable because melatonin is formed from serotonin and, therefore, may be decreased in a BH_4_ deficient state.

Apart from 2 patients with SRD, who were reported to have reduced night time dystonia and improved sleep transition, there is very limited evidence for the use of melatonin in BH_4_Ds [21, 104]. In the 2 studies with low or very low level of evidence, no side effects have been reported.

**R#52 (conditional):** There is a pathophysiological rationale to consider a trial of melatonin in all BH_4_D patients facing sleep induction problems before using other sleep-inducing medications. Prior to this, an optimization of 5-HTP supplementation should be reached (except in AD-GTPCH and PCDD).

### Acute drug treatments

#### Baclofen

Baclofen is a CNS depressant and skeletal muscle relaxant used to treat spasticity. In the literature, there is only one SRD case published in whom baclofen was used. However, no clear description of its clinical effect is provided [104]. No recommendation is possible due to lack of evidence.

**R#53 (GPP):** The application of baclofen could be considered in patients with complications due to spasticity. The decision should be based on individual clinical judgment. See the guideline on diagnosis and management of cerebral palsy in young people [144].

#### Benzodiazepines

Benzodiazepines belong to the broader generally accepted treatment regimen of dystonia. However, for BH_4_Ds, there is no satisfying evidence for the use of benzodiazepines. A single patient with SRD is described who experienced no change in the duration of oculogyric crisis with benzodiazepine treatment [97]. However, the guideline group members describe beneficial effects on prolonged oculogyric crisis in some patients (personal communication).

**R#54 (GPP):** Current evidence for benzodiazepine treatment in BH_4_Ds is very scarce but a treatment attempt can be considered in specific settings, e.g. in sustained oculogyric or dystonic crises, always based on individual clinical judgement.

#### Anti-epileptics

The literature evidence for the use of anti-epileptic treatment in BH_4_Ds is primarily available for DHPRD. The use of various anti-epileptic drugs, most commonly phenobarbital and phenytoin, and always combined with other medications, was reported in 14 cases from 9 studies [93, 94, 145]. Notably, folinic acid supplementation was reported to improve epileptic seizures in DHPRD, too [94, 146, 147]. One patient with SRD treated with valproic acid was reported, but no details regarding the clinical course were provided. For the other BH_4_Ds, the specific antiepileptic drugs used are usually not specified.

**R#55 (GPP):** Epileptic seizures are not a cardinal clinical symptom of BH_4_Ds and should be distinguished by reliable diagnostic approaches from oculogyric crisis or dystonic jerks. If required, any antiepileptic treatment can be used according to the specific indications for different seizure types.

### Other supportive therapies

#### Botulinum toxin injections

Botulinum toxin injections are commonly used to treat focal dystonia. However, since there are only 3 patients with AD-GTPCHD reported in whom botulinum toxin injections were used, there is very limited evidence for its application in BH_4_Ds. There are no patients described with botulinum toxin injections as monotherapy. In one case, botulinum toxin injections were used before the diagnosis of the underlying BH_4_D. 2 other cases were concurrently treated with L-Dopa/DC inhibitor [142, 148], and with trihexyphenidyl in 1 case, which did not resolve dystonic symptoms completely (writer’s cramp, blepharospasm, and retrocollis). All patients improved with botulinum toxin injections; however, the co-administration of other medications does not permit evaluation of the effect of botulinum toxin alone. Side effects were not reported in the literature. Dose and application procedure should follow specific guidelines [149].

**R#56 (conditional):** Botulinum toxin injections should be considered as an option in the case of persistent focal dystonia in AD-GTPCHD if all first- and second-line treatment options have, over an adequate amount of time, shown to be insufficient to control these symptoms. For AR-GTPCHD, DHPRD, PTPSD, SRD, and PCDD, there is no recommendation possible due to lack of evidence.

#### Multidisciplinary treatment

Although there are not sufficient studies or reports on the impact of multidisciplinary treatment in BH_4_Ds available, involvement of a broad team with specialists in physiotherapy, speech therapy, occupational therapy, feeding and nutritional assessment, and (neuro-) psychological treatment should always be part of the complex care provided to BH_4_D patients to improve patient care, prevent secondary complications, and promote neurological development.

#### Psychiatric therapy

There is very limited evidence for the use of psychiatric therapy in the cohort of BH_4_D patients. It is presumed that at least some patients with psychiatric disturbances received psychiatric pharmacotherapy; however, the literature is scarce. There are 2 published cases of adult patients with AD-GTPCHD who received electroconvulsive therapy (ECT) [150, 151]. One of the patients with psychosis [151] developed a neuroleptic malignant syndrome due to haloperidol treatment followed by a prolonged catatonic state, which required ECT. In the other patient, a combination of SSRI and 5-HTP did not prevent the emergence of a delusional depression; he was therefore treated with ECT. ECT therapy had a positive effect in both cases. Side effects of ECT are only mentioned in one case: after the third and fourth treatment sessions, the patient developed postictal disorientation and agitation lasting about 30 min [150]. Overall, no recommendation for a specific psychiatric therapy for the treatment of psychiatric disorders in BH_4_Ds is possible due to lack of evidence.

### Experimental therapies

New experimental therapies are listed at https://clinicaltrials.gov.

### Drugs to avoid in BH_4_ disorders

Drugs with antiemetic and antipsychotic properties, acting as central dopamine antagonists, should be avoided in BH_4_Ds since they have the potential to worsen symptoms of dopamine deficiency [152]. **Metoclopramide** should not be used for the treatment of nausea. **Trimethoprim/sulfamethoxazole** should be avoided because it is reported to cause parkinsonian symptoms in a confirmed patient with DHPRD [146]. This patient was co-treated with L-Dopa/carbidopa and 5-HTP when treatment with trimethoprim-sulfamethoxazole was initiated (folinic acid was added afterwards). The adverse effects reported in this patient were clearly related to the initiation of the antibiotic and their disappearance was related to discontinuation of the treatment [146]. Due to the inhibitory effect of **methotrexate** on DHPR and the interaction with dihydrofolate reductase (DHFR), this treatment may lead to HPA and early neurotoxicity, possibly combined with folate deficiency [153].

### Prenatal treatment

Dopamine signalling is important already for intra-uterine (brain) development [154]. Prenatal oral treatment with L-Dopa/carbidopa to the mother of a genetically confirmed AR-GTPCHD foetus was shown to prevent development of the severe phenotype related to biallelic *GCH1* mutations [68].

**R#57 (research):** Prenatal treatment with levodopa can be beneficial. Since the experience of prenatal treatment in BH_4_Ds is based on single case studies with low to very low evidence, it would be desirable to develop a protocol for further treatment attempts in a controlled and standardized trial.

### Follow-up, transition and special situations

#### Follow-up visits

There are no reports available on standardized follow-up visits in the BH_4_D patient cohort. Therefore, recommendations can only be based on clinical experiences or good clinical practice. Comparable to other inborn errors of metabolism [5, 155], for all BH_4_Ds apart from PCDD life-long, systematic follow-up is recommended to achieve optimal development, to prevent or avoid treatment side-effects, and to evaluate quality of life. In addition, it is not known if and when long-term complications occur. Regular standardized follow-ups allow early identification of patients presenting with such disease related or treatment-related complications.

**R#58 (GPP):** BH_4_D patients should be seen at least yearly by a (child) neurologist with experience in movement disorders or neurometabolic disease, ideally in a multidisciplinary setting. Infants and young children who require frequent dose adjustments during the course of initial dose titration and due to weight gain need to be seen more frequently (e.g. infants every 3 months; older children at least every 6 months)!

The follow-up visits should include the evaluation of:

- **Phe-reduced diet** (if applicable)**:** Daily amount of Phe, intake of amino acid mixture, amino acids in plasma, full blood count, ferritin, parathormone, calcium, phosphate, alkaline phosphatase, vitamin B12
- **Current medication:** Regular intake? Symptoms of over−/underdose?
- **Neurological symptoms:** Motor milestones, seizures, oculogyric crises, vegetative symptoms (sweating, fever, nausea, vomiting, stool frequency, micturition frequency, sleep, behaviour), eating habits, speech development
- **General medical history:** Anthropometric data? Infections? Vaccinations? Narcotics or alcohol abuse?
- Integration and inclusion measures (if applicable)
- Kindergarten, school, education, occupation
- ECG and/or echocardiography (if under treatment with dopamine agonists).
- Neuropsychological development

Although there are no studies or reports on the need for repeated measurement of CSF metabolites as part of ongoing treatment monitoring, CSF analysis of HVA, 5-HIAA, and 5-MTHF can be helpful for drug dose titration or for clarification of otherwise unexplainable clinical irregularities. This is especially true for younger children in whom the spectrum of (neurological) symptoms can be broader or more difficult to assess.

**R#59 (GPP):** Consider CSF analysis of HVA, 5-HIAA, and 5-MTHF for drug dose titration or for clarification of otherwise unexplainable clinical irregularities in all BH_4_Ds apart from PCDD. For the lumbar puncture standard oral treatment should be interrupted as short as possible.

#### Transition

As in many other inborn errors of metabolism, there is a paucity of literature regarding transition from childhood to adulthood in the BH_4_Ds. However, a successful transition to adult care requires the coordinated cooperation of many disciplines. A transitional consultation with the participation of paediatric and adult neurological institutions is very valuable [156]. During the transition process, the following aspects should be considered among others: Role changes of patients, parents and caregivers, active involvement of stakeholders in the planning and decision-making processes, comprehensive knowledge about the illness and its course.

**R#60 (GPP):** Begin planning early for transition of BH_4_D patients to adult care in specialized centers. Multidisciplinary care should be continued.

#### Anaesthesia

Reports on anaesthesia in BH_4_D patients are very scarce in the literature, and do not indicate any particular risks. From the metabolic perspective BH_4_D patients do not require any special precautions and anaesthesia can follow standardized procedures. No specific anaesthetic drugs need to be avoided.

**R#61 (GPP):** Anaesthesia in BH_4_D patients may follow standard protocols. After the operative procedure standard oral treatment should continue as soon as possible.

#### Genetic counselling

On the grounds that BH_4_Ds are inherited metabolic disorders, it is good clinical practice to offer genetic counselling to parents and/or patients. In addition, molecular genetic analysis is the preferred prenatal testing method for all BH_4_Ds **(see R#29)**.

**R#62 (strong):** All patients or parents of patients with BH_4_Ds should be offered standard genetic counselling if available in local care settings.

#### Pregnancy

There are few cases in the literature reporting obstetric and paediatric outcomes in pregnancies of patients with BH_4_Ds [157]. It is important to control disease-related symptoms, adjust the treatment if needed, and monitor the development of the foetus. Therefore, close supervision by a multidisciplinary team (dietitian, (neuro) metabolic consultant, neurologist, gynaecologist, geneticist) is essential during the course of pregnancy and afterwards.

**R#63 (strong):** Intensive supervision during and after the pregnancy by a multidisciplinary team should be provided.

### Patient advocacy groups

Currently, there are the following non-profit volunteer organizations, representing children and families who are affected by a paediatric neurotransmitter disease including BH_4_Ds:

- Pediatric Neurotransmitter Disease Association (www.pndassoc.org) - USA
- Spanish neurotransmitter diseases association “De neu” (www.deneu.org) - Spain
- German group for patients and parents with all kinds of neurotransmitter related disorders (www.dig-pku.de/wcf/index.php?neurotransmitterstoerungen-nts/) - Germany
- Organization to support families with children suffering from neurotransmitter diseases (www.hrabrisa.rs/en/) - Serbia

Regular updates on patient advocacy groups can be found under https://intd-online.org/patients/.

## Conclusion

This is the first consensus guideline for the diagnosis and management of BH_4_ deficiencies. All recommendations are based on the available literature evidence and were phrased in a transparent consensus process by the iNTD guideline working group. The guideline is intended for clinicians, metabolic biochemists and paramedical specialists involved in the care of patients with BH_4_ deficiencies. It will help to harmonize clinical practice and to standardize and improve care for BH_4_ deficient patients.

## Supplementary information

**Additional file 1: Table S1.** Key questions for the Guideline on diagnosis and treatment of BH_4_ deficiencies.

**Additional file 2: Figure S2.** Flow chart showing the systematic literature search, and number and type of included sources.

## Data Availability

The datasets used and/or analysed during the current study are available from the corresponding authors on reasonable request.

## Abbreviations

7,8-BH_2_: 7,8-dihydrobiopterin
5-HIAA: 5-hydroxyindoleacetic acid
5-HTP: 5-hydroxytryptophan
5-MTHF: 5-methyltetrahydrofolate
3-MT: 3-Methoxytyramine
3-OMD: 3-O-methyldopa
6-PTPS: 6-pyruvoyltetrahydropterin synthase
AA: Amino acids
AADCD: Aromatic l-amino acid decarboxylase deficiency
AD: Autosomal dominant
AGMO: Alkylglycerol mono-oxygenase
AR: Autosomal recessive
BBB: Blood-brain-barrier
Bio: Biopterin
BH_2_: Dihydrobiopterin
BH_4_: Tetrahydrobiopterin
BW: Body weight
CNS: Central nervous system
CSF: Cerebrospinal fluid
CCT: Cranial computer tomography
CMRI: Cranial magnetic resonance imaging
COMT: Catechol-O-methyl transferase
DA: Dopamine agonist
DBS: Dry blood spot
DC: Decarboxylase
DCI: Decarboxylase inhibitor
DHFR: Dihydrofolate reductase
DHPG: 3,5-dihydroxyphenylglycine
DHPR: q-dihydropteridine reductase
DHPRD: Dihydropteridine reductase deficiency
DOPAC: 3,4-Dihydroxyphenylacetic acid
DRD: Dopa-responsive dystonia
DT: Dopamine transporter
ECT: Electroconvulsive therapy
EEG: Electroencephalography
GRADE: Grading of Recommendations, Assessment, Development and Evaluation
GTPCH: Guanosine triphosphate cyclohydrolase I
GTPCHD: Guanosine triphosphate cyclohydrolase I deficiency
GPP: Good Practice Point
HPA: Hyperphenylalaninemia
HVA: Homovanillic acid
iNTD: International Working Group on Neurotransmitter Related Disorders
L-Dopa: l-3,4-dihydroxyphenylalanine
MAO(−I): Monoamine oxidase (inhibitor)
MHPG: 3-Methoxy-4-hydroxyphenylglycol
MLPA: Multiple ligation-dependent probe amplification
MS: Mass spectrometry
NBS: Newborn screening
NGS: Next-generation sequencing
Neo: Neopterin
NOS: Nitric oxide synthase
MODY: Maturity Onset Diabetes of the Young
PAH: Phenylalanine hydroxylase
PCD: Pterin-4-alpha-carbinolamine dehydratase
PCDD: Pterin-4-alpha-carbinolamine dehydratase deficiency
Phe: Phenylalanine
PKU: Phenylketonuria
Prim: Primapterin
PTPSD: 6-pyruvoyltetrahydropterin synthase deficiency
SAM: s-adenosyl-methionine
SIGN: Scottish Intercollegiate Guideline Network
SR: Sepiapterin reductase
SRD: Sepiapterin reductase deficiency
SSRI: Selective serotonin reuptake inhibitors
TH: Tyrosine hydroxylase
TPH: Tryptophan hydroxylase
Tyr: Tyrosine
VLA: Vanillactic acid
VMA: Vanillylmandelic acid
