# Correction to: Consensus guideline for the diagnosis and treatment of tetrahydrobiopterin (BH4) deficiencies

**DOI:** 10.1186/s13023-020-01464-y

**Published:** 2020-08-05

**Authors:** Thomas Opladen, Eduardo López-Laso, Elisenda Cortès-Saladelafont, Toni S. Pearson, H. Serap Sivri, Yilmaz Yildiz, Birgit Assmann, Manju A. Kurian, Vincenzo Leuzzi, Simon Heales, Simon Pope, Francesco Porta, Angeles García-Cazorla, Tomáš Honzík, Roser Pons, Luc Regal, Helly Goez, Rafael Artuch, Georg F. Hoffmann, Gabriella Horvath, Beat Thöny, Sabine Scholl-Bürgi, Alberto Burlina, Marcel M. Verbeek, Mario Mastrangelo, Jennifer Friedman, Tessa Wassenberg, Kathrin Jeltsch, Jan Kulhánek, Oya Kuseyri Hübschmann, on behalf of the International Working Group on Neurotransmitter related Disorders (iNTD)

**Affiliations:** 1 Division of Child Neurology and Metabolic Disorders, University Children’s Hospital, Heidelberg, Germany; 2 Pediatric Neurology Unit, Department of Pediatrics, University Hospital Reina Sofía, IMIBIC and CIBERER, Córdoba, Spain; 3 Inborn errors of metabolism Unit, Institut de Recerca Sant Joan de Déu and CIBERER-ISCIII, Barcelona, Spain; 4 Unit of Pediatric Neurology and Metabolic Disorders, Department of Pediatrics, Hospital Germans Trias i Pujol, and Faculty of Medicine, Universitat Autònoma de Barcelona, Badalona, Spain; 5 Department of Neurology, Washington University School of Medicine, St. Louis, USA; 6 Department of Pediatrics, Section of Metabolism, Hacettepe University, Faculty of Medicine, 06100 Ankara, Turkey; 7 Developmental Neurosciences, UCL Great Ormond Street-Institute of Child Health, London, UK; 8 Department of Neurology, Great Ormond Street Hospital, London, UK; 9 Unit of Child Neurology and Psychiatry, Department of Human Neuroscience, Sapienza University of Rome, Rome, Italy; 10 Neurometabolic Unit, National Hospital, Queen Square, London, UK; 11 Department of Pediatrics, AOU Città della Salute e della Scienza, Torino, Italy; 12 Department of Paediatrics and Adolescent Medicine, First Faculty of Medicine, Charles University and General University Hospital in Prague, Prague, Czech Republic; 13 First Department of Pediatrics of the University of Athens, Aghia Sofia Hospital, Athens, Greece; 14 Department of Pediatric, Pediatric Neurology and Metabolism Unit, UZ Brussel, Brussels, Belgium; 15 Department of Pediatrics, University of Alberta Glenrose Rehabilitation Hospital, Edmonton, Canada; 16 Clinical biochemistry department, Institut de Recerca Sant Joan de Déu, CIBERER and MetabERN Hospital Sant Joan de Déu, Barcelona, Spain; 17 Department of Pediatrics, Division of Biochemical Genetics, BC Children’s Hospital, University of British Columbia, Vancouver, BC Canada; 18 Division of Metabolism, University Children’s Hospital Zurich, Zürich, Switzerland; 19 Clinic for Pediatrics I, Medical University of Innsbruck, Anichstr 35, Innsbruck, Austria; 20 U.O.C. Malattie Metaboliche Ereditarie, Dipartimento della Salute della Donna e del Bambino, Azienda Ospedaliera Universitaria di Padova - Campus Biomedico Pietro d’Abano, Padova, Italy; 21 Departments of Neurology and Laboratory Medicine, Alzheimer Centre, Radboud University Medical Center, Donders Institute for Brain, Cognition and Behaviour, Nijmegen, The Netherlands; 22 UCSD Departments of Neuroscience and Pediatrics, Rady Children’s Hospital Division of Neurology, Rady Children’s Institute for Genomic Medicine, San Diego, USA

**Correction to: Orphanet Journal of Rare Diseases 15, 126 (2020)**

**https://doi.org/10.1186/s13023-020-01379-8**

Following the original article's publication [1] the authors asked for the correction of Fig. 2, since the names of the disease genes [*GCH1* and *PCBD1*] in the figure published did not match the listed diseases [AR-GTPCHD and PCDD]. The correct Fig. 2 is shown below:

**Fig. 2:**
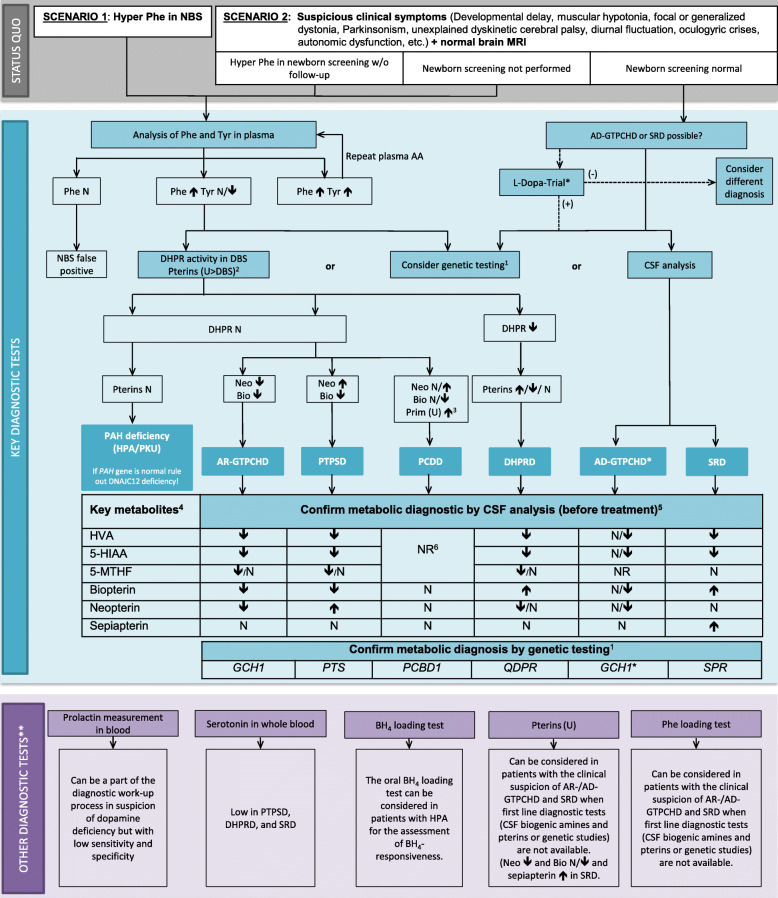
Diagnostic flowchart for differential diagnosis of BH_4_Ds with and without HPA. ^1^Consider genetic HPA workup depending on availability and financial resources. The gene panel should include the *QDPR, GCH1, PTS PCBD1, SPR* genes as well as *DNAJC12.* For *GCH1*, consider MLPA if Sanger sequencing is negative. ^2^The analysis in urine is more sensitive than in DBS and pathological patterns suggestive for PCDD and SRD can only be detected in urine but not in DBS. ^3^Primapterin measurement in urine is only elevated in PCDD. ^4^Aminoacids in CSF are not required for diagnosis of BH_4_Ds. ^5^CSF analysis should always include standard measurements (cell count, proteins, glucose and lactate). ^6^Recommendation against measurements of HVA, 5-HIAA, 5-MTHF, and pterins in CSF in the case of PCDD. (*) A diagnostic L-Dopa trial should be limited to children with symptoms suggestive of dopa-responsive dystonia or to situations where biochemical and genetic diagnostic tools are not available. If the diagnostic L-Dopa trial is positive but the results of CSF biochemical and/or molecular genetic testing are not compatible with AD-GTPCHD or SRD, further aetiologies for dopa responsive dystonia should be considered (e.g. juvenile parkinsonism (PARK2gene)). (**) Can be considered if available. See text for more detailed information. Abbreviations: 5-HIAA, 5-hydroxyindoleacetic acid; 5-MTHF, 5-methyltetrahydrofolate; AA: amino acids; AD−/AR- GTPCHD: guanosine triphosphate cyclohydrolase I deficiency; BH_4_, tetrahydrobiopterin; Bio: biopterin; CSF: cerebrospinal fluid; DBS: dry blood spot; DHPR: q-dihydropteridine reductase; DHPRD, dihydropteridine reductase deficiency; HVA, homovanillic acid; MRI, magnetic resonance imaging; N: normal; NBS: newborn screening; Neo: neopterin; NR: not reported; PAH: phenylalanine hydroxylase; Phe: phenylalanine; PKU: phenylketonuria; Prim: primapterin; PTPSD, 6-pyruvoyltetrahydropterin synthase deficiency; SRD: sepiapterin reductase deficiency; Tyr: tyrosine; u: urine; (+) = positive effect; (−) = no or no clear effect

In the context of the manuscript correction and in order to match the text content, the words "apart from DHPRD" should be removed from the second row and second column of Table 4, as shown below:

**Table 4:** Recommended drugs and doses for BH_4_ disorders

	Disorder	Starting dose	Doses	Target dose	Maximum dose	Management suggestion	Comment
**First line treatments**							
**Phe-reduced diet**	All BH_4_D with HPA					Titrate Phe restriction according to Phe levels in DBS or plasma	Follow PKU national treatment recommendations Use either Phe reduced diet or Sapropterin dihydrochloride to control Phe levels
**Sapropterin dihydrochloride**	All BH_4_D with HPA	2-5 mg/kg BW/day	Divided in 1–3 doses/ day	5–10 mg/kg BW/day	20 mg/kg BW/day	Titrate dose according to Phe levels in DBS or plasma	Follow PKU national treatment recommendations Use either Phe reduced diet or Sapropterin dihydrochloride to control Phe levels
**L-Dopa/DC inhibitor (carbidopa/benserazide) 4:1**	All BH_4_D apart from PCDD	0.5 mg–1 mg/kg BW/day Dose recommendation relates to L-Dopa component!	Divided in 2–6 doses/ day	AD-GTPCHD: 3–7 mg/kg BW/day All other BH_4_D: 10 mg/kg BW/day or maximally tolerated dosage Dose recommendation relates to L-Dopa component!	Depending on clinical symptoms. Some patients need more than 10 mg/kg BW/day for resolving clinical symptoms	Increase 0.5–1 mg/kg BW/day per week Follow BW adaption until the BW of 40 kg. After 40 kg adjust depending on clinical symptoms Consider analysis of CSF HVA for dose adjustment	In young infants at least as many dosages as meals would be ideal (usually 5–6 /day)
**5-Hydroxytryptophan (5-HTP)**	All BH_4_D apart from AD-GTPCHD and PCDD	1–2 mg/kg BW/day	Divided in 3–6 doses/day	Published target dose recommendations are highly variable 5-HTP doses are usually lower than L-Dopa doses		Titrate slowly (1–2 mg/kg BW/day per week) depending on clinical picture and side effects Consider analysis of CSF 5HIAA for dose finding	5-HTP should follow L-Dopa/DCI treatment initiation Always in combination with a peripheral decarboxylase inhibitor (for example by simultaneous application with L-Dopa/DC inhibitor)
**Folinic acid**	In DHPRD and all BH_4_D with low 5-MTHF in CSF		Divided in 1–2 doses/day	10–20 mg/day		No titration needed Consider analysis of CSF 5MTHF for dose finding	
**Second line treatments**							
**Pramipexole**^**a**^ (Dopamine agonist)	All BH_4_D apart from PCDD	3.5–7 μg/kg/BW/day (base) 5–10 μg/kgBW/day (salt) Note: Distinction in salt and base content! (see product insert)	Divided in 3 equal doses/day	Titrate to clinical Symptoms	75 μg/kg BW/day (3.3 mg/d base / 4 mg/d salt)	Increase every 7 days by 5 μg/kg BW/d	
**Bromocriptine**^**a**^ (Dopamine agonist)	All BH_4_D apart from PCDD	0.1 mg/kg BW/day	Divided in 2–3 doses/day	Titrate to clinical Symptoms	0.5 mg/kg/d (or 30 mg/d)	Increase every 7 days by 0.1 mg/kg BW/d	
**Rotigotine**^**a**^ (transdermal dopamine agonist)	All BH_4_D apart from PCDD	2 mg/day		Titrate to clinical Symptoms	8 mg/day	Increase weekly by 1 mg	Children > 12 years Exchange patch every 24 h
**Selegiline**^**a**^ (MAO B inhibitor)	All BH_4_D apart from PCDD	0.1 mg/kg BW/day	Divided in 2 (−3) doses/day	Titrate to clinical Symptoms	0.3 mg/kg/d (or 10 mg/d)	Increase every 2 weeks by 0.1 mg/kg BW/d	Can cause sleep disturbances – morning and afternoon or lunchtime dosage is possible ATTENTION: orally disintegrating preparation needs much less dosage because the first-pass effect of the liver is avoided
**Third line treatments**							
**Trihexyphenidyl**^**a**^ (Anticholinergic drugs)	All BH_4_D apart from PCDD	< 15 kg: start 0.5–1 mg/day > 15 kg: start 2 mg/day	< 15 kg: in 1 dose > 15 kg: in 2 doses	Effective dose highly variable (6–60 mg) Titrate to clinical Symptoms	Maximum dose: < 15 kg BW 30 mg/day > 15 kg BW 60 mg/d	Increase every 7 days by 1–2 mg/d in 2–4 doses/d	Consider side effects: like dry mouth, dry eyes, blurred vision (mydriasis), urine retention, constipation.
**Entacapone**^**a**^ (COMT inhibitor)	All BH_4_D apart from PCDD	200 mg (adult)			Up to 2.000 mg		In many countries licensed only for adults. Comedication with L-Dopa/DC inhibitor Consider reduction of concomitant L-Dopa supplementation (10–30%)
**Sertaline**^**a**^ (SSRI)	All BH_4_D apart from PCDD	6–12 years: 25 mg/day in 1 dose > 12 years: 50 mg/day in 1 dose	6–12 years: in 1 dose > 12 years: in 1 dose	Children 50 mg/day	50 mg/day < 12 years 200 mg/day > 12 years	6–12 years: increase after 7 days to 50 mg/day in 1 dose > 12 years 50 mg/day in 1 dose	Don’t stop treatment suddenly Note: Elevated risk of serotonin syndrome (SS) or malignant neuroleptic syndrome (MNS) when used with drugs impacting serotonergic pathway (e.g. 5-HTP, MAO inhibitors)
**Melatonin**^**a**^	All BH_4_D apart from PCDD	0.01–0.03 mg/kg/day			5–8 mg/day		Slow release preparation for sleep-maintenance insomnia available in some countries

Please note: The doses given are in a range typically used and have been published. In individual patients, some adjustment may be necessary depending on symptom response and side effects

^a^The evaluated literature did not provide BH_4_D specific treatment dose recommendations for this drug. The listed doses, therefore, indicate treatment recommendations from Summary of Product Characteristics (SmPC) or neurotransmitter related publications (e.g. [119])

*Abbreviations*: *5-HIAA* 5-hydroxyindoleacetic acid, *5-HTP* 5-hydroxytryptophan, *5-MTHF* 5- methyltetrahydrofolate, *HVA* Homovanillic acid, *AD-GTPCHD* Autosomal-dominant guanosine triphosphate cyclohydrolase I deficiency, *BH*_*4*_*D* Tetrahydrobiopterin deficiency, *BW* Body weight, *COMT* Catechol-O-methyl transferase, *CSF* Cerebrospinal fluid, *DBS* Dry blood spot, *DC* Decarboxylase, *DCI* Decarboxylase inhibitor, *DHPRD* Dihydropteridine reductase deficiency, *L-Dopa* L-3,4-dihydroxyphenylalanine, *MAO B* Monoamine oxidase B, *PCDD* Pterin-4-alpha-carbinolamine dehydratase deficiency, *Phe* Phenylalanine, *PKU* Phenylketonuria, *SSRI* Selective serotonin reuptake inhibitor
