# Supplementary material for: Consensus guideline for the diagnosis and treatment of tetrahydrobiopterin (BH4) deficiencies
Source: Orphanet J Rare Dis. 2020 May 26;15:126. doi: 10.1186/s13023-020-01379-8 (PMC7251883; doi:10.1186/s13023-020-01379-8)
Supplement: Supplementary file 1 — Additional file 1: Table S1. Key questions for the Guideline on diagnosis and treatment of BH4 deficiencies. [file 13023_2020_1379_MOESM1_ESM.docx]

**Key questions for the Guideline on diagnosis and treatment of BH_4_ deficiencies**

Part I: Diagnosis

1. Clinical presentation:
   1. What neurological signs and symptoms are described in BH_4_ deficiencies before treatment?
      1. Movement disorder (e.g. tone regulation problems, delayed milestones, parkinsonism)
      2. Speech development
      3. Cognitive development
      4. Behavioural problems
      5. Sleeping problems
      6. Epilepsy
      7. Psychiatric problems
      8. ADHs
      9. Other
   2. What non-neurological signs and symptoms are described in BH_4_ deficiencies before treatment?
      1. Cardiovascular
      2. Ear-nose-throat
      3. Gastro-intestinal (feeding?)….
      4. Metabolic
      5. Endocrine
      6. Adipositas
      7. Other
   3. What is the time of presentation of first symptoms?
   4. What is the age of diagnosis?
   5. What is the clinical spectrum of BH_4_ deficiencies?
      1. Level of functioning (described how?) before treatment?
2. Laboratory diagnosis:
   1. What is the diagnostic value of Newborn screening?
   2. What is the diagnostic value of pterins in
      1. DBS
      2. Urine
      3. Phenylalanine loading test
   3. What is the diagnostic value of DHPR in DBS
   4. Which diagnostic tests are available and what are their diagnostic values?
      1. Lumbar puncture
         1. What measurements should be included?
            1. Neurotransmitter metabolites:

HVA/5-HIAA/VMA/3-OMD

Other

- - - - 1. Pterines
        2. 5MTHF
        3. Pyridoxal phosphate
        4. Amino acids
        5. Sepiapterin
        6. Melatonin
        7. Other
      1. What is the recommended handling of CSF?
      2. What is the recommended method of measurements?
      3. In which countries/ centres can this test be performed?
    1. Enzyme activity measurement in plasma/ Erythrocytes/ Fibroblasts
       1. What is possible?
       2. What is the recommended method?
       3. In which countries/ centres can this test be performed?
    2. Genetic diagnosis
       1. Method/ handling
       2. List of mutations?
       3. In which countries/ centres can this test be performed?
    3. Blood tests:
       1. Prolactine
          1. What is the diagnostic value of prolactin in BH_4_ deficiencies?
       2. Serotonin (whole blood)
       3. Phenylalanine (plasma and DBS)
       4. Phenylalanien loading test
  1. What is the best screening test for BH_4_ deficiencies? (handling, reference values)
  2. What are mandatory tests for definite diagnosis of BH_4_ deficiencies?
  3. What is the value of MR, CCT, Fluordopa PET imaging of the brain?
  4. What is known about panel diagnostics or whole exome sequencing?

**Statement: proposed diagnostic algorithm for BH_4_ deficiencies**

1. Genotype/ Phenotype correlations
   1. Are there genotype/ biochemical and/or clinical phenotypes correlations in BH4 deficiencies?
      1. Pterine measurements in DBS and urine
      2. CSF measurements
      3. Genetic analysis
      4. Response to treatment

Part II: Treatment

1. What is the evidence for the different options of maintenance drug therapy, including dosage and side effects?
2. For each of the different drugs: is there a subset of patients for which this therapy has better or worse outcome?
   1. Phe reduced diet
   2. Sapropterin
   3. Levodopa
      1. Carbidopa
   4. 5-hydroxytryptophan
   5. Dopamine agonists
      1. Bromocriptine
      2. Pramipexol
      3. ....
   6. MAO-inhibitors
      1. Selective
      2. Non-selective
   7. Anti-cholinergic agents
   8. Alfa-adrenoreceptor agonists
   9. Folinic acid
   10. Melatonin
   11. BH_2_
   12. Selective serotonin reuptake inhibitors
   13. Other
       1. Benzodiazepines,
       2. Botulintoxin
       3. Other
3. Is there any recommendable non- medical treatment?
   1. Physiotherapy
   2. Logopaedic treatment
   3. Occupational treatmentietary treatment?
4. Acute drug therapy
   1. Baclofen
   2. Benzodiazepine
   3. Chloralhydrate
   4. Phenobarbiton?
5. Surgical treatment options / deep brain stimulator?
   1. Gene therapy
6. Concomitant medication
   1. Antiepileptic therapy, are there recommendations on drugs of choice?
   2. Psycatric therapy/ treatment
7. Contraindication Are there any drugs to avoid in BH_4_ deficiencies?

**Statement: proposed therapeutic algorithm for BH_4_-deficiencies**

Part III: Standard long-term management of BH4 patients

1. Topics that should be covered on follow up visits
2. Monitoring:
   1. Nutrional status (bone, vitamins, iron, selenium, zinc, vitamin D?)
   2. Anthropometric data
   3. Growth
   4. Video documentation
   5. Which lab tests should be performed for the monitoring after diagnosis?
3. Prolactin (blood)
4. CSF
5. Specific biochemical monitoring (lumbar puncture?)
6. (Neuro) Radiological monitoring (brain MRI, RxhHipp)

**Statement: proposed long-term management algorithm for BH_4_ deficiencies**

Part IV: What are known complications in BH4 deficiencies despite adequate treatment (caused by disease/ caused by treatment?)

- Hypoglycemia?
- Cardiac decompensation?
- Orthopaedic complication (contractures, luxation…)
- Sleeping problems
- Dyskinesia
- Risk of Parkinsons disease
- ID
- Other organ complications known?

Part V: Which aspects are to consider regarding transition and social issues

- Behavioural problems
- Psychiatric problems e.g. depression
- Concentration problems

Part V: Special situations

1. Anesthesia
2. Intensive care
3. Prenatal diagnosis
4. Pregnancy
5. Infections
6. Vaccinations
