# Supplementary figures and images for: Consensus guideline for the diagnosis and treatment of tetrahydrobiopterin (BH4) deficiencies
Source: Orphanet J Rare Dis. 2020 May 26;15:126. doi: 10.1186/s13023-020-01379-8 (PMC7251883; doi:10.1186/s13023-020-01379-8)

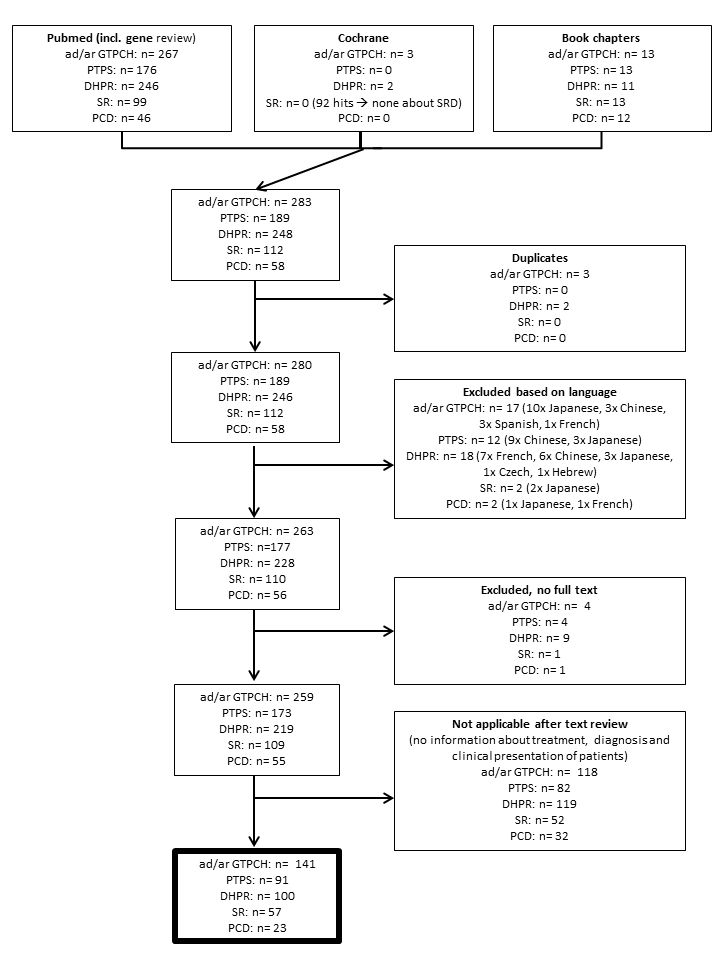

Supplement: Supplementary file 2 — Additional file 2: Figure S2. Flow chart showing the systematic literature search, and number and type of included sources. [file 13023_2020_1379_MOESM2_ESM.tif]
